# Risk factors for scabies, tungiasis, and tinea infections among schoolchildren in southern Ethiopia: A cross-sectional Bayesian multilevel model

**DOI:** 10.1371/journal.pntd.0009816

**Published:** 2021-10-06

**Authors:** Hiwot Hailu Amare, Bernt Lindtjorn

**Affiliations:** 1 School of Public Health, College of Medicine and Health Sciences, Hawassa University, Hawassa, Ethiopia; 2 Centre for International Health, University of Bergen, Bergen, Norway; 3 Department of Public Health, College of Health Sciences and Medicine, Dilla University, Dilla, Ethiopia; Federal University of Agriculture Abeokuta, NIGERIA

## Abstract

**Background:**

Skin problems cause significant sickness in communities with poor living conditions, but they have received less attention in national or global health studies because of their low mortality rates. In many developing regions, the prevalence of parasitic skin diseases among schoolchildren is not reported. Previous studies thus have attempted to identify risk factors for these conditions using the frequentist approach. This study aimed to assess the occurrence and risk factors of skin infections among rural schoolchildren in southern Ethiopia by combining a frequentist and a Bayesian approach.

**Methodology/Principal findings:**

Using three-stage random sampling, we assessed 864 schoolchildren aged 7–14 years from the Wonago district in southern Ethiopia. We detected potential risk factors for scabies, tungiasis, and tinea infections and recorded their hygienic practices and socio-demographic information. The frequentist model revealed a clustering effect of 8.8% at the classroom level and an insignificant effect at the school level. The Bayesian model revealed a clustering effect of 16% at the classroom level and 5.3% at the school level. Almost three-fourths of the sample had at least one type of skin problem, and boys were at higher overall risk than girls (adjusted odds ratio [aOR] 1.55 [95% Bayesian credible interval [BCI] 1.01, 2.28). Risk factors included unclean fingernails (aOR 1.85 [95% BCI 1.08, 2.97]); not washing the body (aOR 1.90 [95% BCI 1.21, 2.85]) and hair (aOR 3.07 [95% BCI 1.98, 4.57]) with soap every week; sharing a bed (aOR 1.97 [95% BCI 1.27, 2.89]), clothes (aOR 5.65 [95% BCI 3.31, 9.21]), or combs (aOR 3.65 [95% BCI 2.28, 5.53]); and living in a poor household (aOR 1.76 [95% BCI 1.03, 2.83]). Washing legs and feet with soap daily was identified as a protective factor for each of the three skin diseases (aOR 0.23 [95% BCI 0.15, 0.33]).

**Conclusions/Significance:**

We observed high variation in skin problems at the classroom level, indicating the presence of shared risk factors in these locations. The findings suggest the need to improve children’s personal hygiene via health education by schoolteachers and health workers.

## Author summary

Skin problems are common disorders in resource poor settings. Scabies and tungiasis are neglected tropical diseases causing significant sickness in communities with poor living conditions. Scabies is dermatosis caused by a burrowing mite, *Sarcoptes scabiei var*. *hominis*. Tungiasis is caused by a flea, *Tunga penetrans*, infesting the skin, usually on the feet or hands. Tinea infection is a fungal dermatophyte infection of the skin and the most common dermatological problem among schoolchildren in Ethiopia, especially tinea capitis. Application of a Bayesian approach to identify possible risk factors for these problems has seldom been used. We found that compared to the frequentist model, the Bayesian model better explained school- and classroom-level variations in skin problems among schoolchildren. Our findings also suggest that transmission of skin infections, especially fungal and scabies infections, frequently occurs in schools and classrooms. We identified several risk factors for these conditions, including low socioeconomic status; unclean fingernails; not washing with soap at least every week; and sharing beds, clothes, and combs. Thus, it is important to improve the personal hygiene of schoolchildren through education. Regular checkups by teachers also can improve skin health and related morbidity in rural schoolchildren in southern Ethiopia.

## Introduction

Skin problems are major causes of disability worldwide. In 2013, the estimated disability-adjusted life years for skin problems was 1.79% of the total global burden of disease, including 0.15% for fungal skin diseases and 0.07% for scabies [1]. The prevalence of tungiasis (Jigger flea) infections is estimated to be up to 50% in some endemic areas [2]. Unfortunately, skin problems have received less attention in the national or global health studies because of their low mortality [3]. This study thus aimed to assess the occurrence and risk factors of three parasitic skin diseases (scabies, tungiasis, and tinea) and their risk factors among rural schoolchildren in southern Ethiopia using both a frequentist and Bayesian approach.

Neglected parasitic skin diseases are important medical challenges in resource-poor settings [4, 5]. Scabies, an intensely itchy ectoparasitic skin disease, results from infestations of the mite *Sarcoptes scabiei var*. *hominis* [5, 6]. Scabies lesions can occur in several body sites, but the topographic distribution of scabies lesions may vary with age [6–9]. Globally, it affects over 200 million people, with prevalence ranging from 0.2% to 71% [10]. Half of school-aged children in southern Ethiopia have scabies [11]. Tungiasis is a parasitic skin disease caused by the female sand flea *Tunga penetrans*, also known as the jigger flea [12]. It causes erythema, edema tenderness, itching, and pain in acute phase; and shining skin, desquamation, nail and toes deformity or loss of nails may occur in the chronic phase [13]. It have also socio-economic impact that includes difficulty walking or gripping, sleep disturbances, stigma, and all of which affects quality of life, school absenteeism, and dropout rates [13–16]. Bacterial superinfection is almost constant. Death may ensue when bacterial superinfection causes tetanus, gangrene, or septicaemia [13, 14, 17]. Ethiopia has a favorable environment for jigger fleas [18], and the prevalence of tungiasis ranges between 35% to 60% among school-aged children in southern Ethiopia [16, 19]. Tinea is a fungal infection caused by dermatophyte infestation, usually on the scalp, foot, hand, groin, nails, or face [20]. A recent study has indicated that one in five children in Africa has tinea capitis [21], and more than 96% (132.6 million) of cases occur in sub-Saharan Africa, mostly in Nigeria and Ethiopia [21]. The prevalence among schoolchildren in southern Ethiopia is about 25% [22]. Poor hygiene, large families, overcrowding, shared fomites, poverty, and environmental conditions are important underlying social determinants for these three diseases.

Schoolchildren in Ethiopia are vulnerable to various health problems [23]. High rates of nutritional disorders, intestinal parasitic infections, and skin problems have been reported [24]. Although some school health programs include programs to address scabies, tungiasis, and fungal infections, including mandatory periodic screenings, most of these programs are non-functional [23]. In 2012, Ethiopia established the National School Health and Nutrition Strategy aiming to improve schoolchildren’s access to health interventions [25], such as nutrition [26], deworming [27], and water, sanitation, and hygiene [24]. These efforts are ongoing, but major gaps persist in the implementation of these school health services [23].

Most studies on skin problems among primary schoolchildren in Ethiopia did not assess them collectively [19, 22, 28, 29] or focus on scabies [11, 30–33]. To our knowledge, reports of scabies, tungiasis, and tinea prevalence among schoolchildren in southern Ethiopia have not been compiled independently. Moreover, previous studies on these diseases in Ethiopia have used a frequentist approach to identify risk factors. We examine whether a Bayesian model, which estimates credible intervals for unknown parameters from posterior distribution [34, 35], can provide important and additional information for assessing and managing these diseases.

## Methods

### Ethics statement

The institutional review board at the College of Medicine and Health Sciences of Hawassa University (IRB/005/09) and the Regional Ethical Committee of Western Norway (2016/1900/REK vest) provided ethical clearance. The Gedeo Zone Health Department and District Education Office provided a letter of permission. School directors and teachers were informed about the study objectives and procedures. We obtained informed written (signed) and verbal (thumb print) consent from all study participants’ parents or guardians and written permission (assent) from children aged 12 years and older before the interviews. The participants’ privacy and confidentiality were maintained. Children diagnosed with scabies and tinea infections were referred to the nearest health institution for treatment according to the standard national guidelines [36]. At the end of the study, we provided soap and Vaseline ointment to each child to encourage them to protect their legs and feet. Health education on personal hygiene also was provided to all children in each school.

### Study location, design, participants, and sample size

We conducted this cross-sectional survey as part of a large project examining anemia, stunting, and intestinal helminth infections among schoolchildren in the Wonago district of the Gedeo zone in southern Ethiopia [37, 38]. The study period was from February 2017 to June 2017. The survey area, design, and sample size estimation are described in detail elsewhere [37, 38]. The study population included 864 randomly recruited schoolchildren aged 7–14 years and their parents or guardians. Using a three-stage cluster sampling method, we first randomly selected four schools and then 24 classrooms totaling 2,384 children. Children aged below 7 years and older than 14 years were excluded from this study. Finally, we included 864 children. Children were recruited at their schools, and their parents or guardians were contacted by visiting their homes. S1 Fig. illustrates the recruitment process.

### Data collection tools and procedures

Using ten trained enumerators, we conducted interviews via a structured and pretested questionnaire, which was adapted and developed in English and then translated into the local Gedeo language. All personnel who participated in data collection, supervision, and data entry were trained accordingly. We conducted interviews with children at their schools and with the parents or guardians in their homes. We conducted a pre-test to validate the measurement tools; to reduce potential bias, the pre-test included 42 children from another primary school not included in this study. The completeness and consistency of data were regularly checked by supervisors.

Information related to the child’s personal hygiene and household factors was collected via interviews and observation. Individual exposure variables included sex, age, personal hygiene (frequency of washing body, legs, feet, and hair with soap; frequency of nail trimming; presence of unclean fingernails; habit of walking barefoot; presence of footwear at time of examination; sharing of beds, combs, and clothes). The education level of the mother and father were included as parents’ exposure variables. Household factors included family size and wealth status. Apart from access to health education on personal hygiene, school-level factors included information on availability of drinking water, handwashing facilities, latrines, and toilet paper.

### Diagnosis and measurement of skin problems

Physical examinations from scalp to toes were performed in daylight in a private place by one of three nurses (a male nurse for boys and female nurse for girls) experienced in clinical diagnoses of dermatologic cases. Children’s feet and hands were cleaned with water and soap before diagnosis of tungiasis. Any identified or suspected skin problems were recorded according to the involved site. To minimize bias in these assessments, 10% (86) of children were examined by two nurses to ensure reproducibility of results. In cases of disagreement, a third and experienced examiner joined the diagnostic process to reach a consensus.

The outcome variable for this study was the presence or absence of scabies, tungiasis, or tinea infection. Scabies was diagnosed via clinical findings of typical skin lesions on the hands, interdigital places, wrists, arms, elbows, armpits, abdomen, chest, mamillar or perimammillar areas, back, buttocks, genitalia, legs, or feet; as well as a history of itching that intensified at night [30, 39, 40]. Tinea infections were classified according to the involved body site [20, 41–44]: tinea capitis (scalp), tinea corporis (torso, arms, and legs), tinea pedis (feet), tinea cruris (groin), tinea unguium (nails), tinea faciei (face), and tinea manuum (hands). Tinea capitis involved one or more scaly patches of alopecia with hairs broken at the skin line (black dots) and crusting. Tinea corporis presented as a red or hyperpigmented lesions on skin other than the face, scalp, groin, hands, or feet. Tinea pedis involved red, scaly, fissures of the foot and maceration and itching between the toes extending to the sole borders, with occasional involvement of the dorsum. Tinea cruris was diagnosed in cases involving the groin and upper thigh but sparing the scrotum and penis. Yellow-brown to black discoloration of the distal portion of the nail, proximal nail bed, or undersurface of the nail plate was diagnosed as tinea unguium. Typical red or pink hyperpigmentation or lesions on the face was diagnosed as tinea faciei. Tinea manuum presented as a red and scaly lesions on the palmar or dorsum of the hands [20, 41–44]. Tungiasis was diagnosed with Fortaleza classification via clinical findings of penetrating sand fleas: a small, dark, itchy spot in the epidermis with visible posterior parts of the parasite, with or without local pain; a brownish or black dot surrounded by erythema; a circular yellow-white patch with a central black dot representing the posterior segments of the parasite; a circular brownish-black crust with or without surrounding necrosis of the epidermis. We also recorded a manipulated lesions: a characteristic crater-like sore or lesion and suppurating lesions caused by the use of non-sterile perforating instruments (i.e. needles) [45].

### Statistical methods

Epi-data version 3.1 (EpiData Association; Odense Denmark, 2004) was used for double-data entry. Prior to analysis, data were cleaned, missing values addressed, and then data exported to SPSS version 20 (IBM Corp, 2011) and STATA 15 software (StataCorp LLC, College Station, TX, 2017) for analysis. Study variables were described using frequencies, percentages, means, ranges, and standard deviations (SD). We applied cross tabulation to calculate the percentage of categorical variables in relation to outcome variables for any scabies, tungiasis, and tinea infections. Using principal component analysis [46], we constructed a wealth index using 14 household asset variables described elsewhere in detail [37]. The socioeconomic indictors were categorized into poor, middle, and rich households based on the first component explaining 28.3% of the variance in the data. A frequentist model and a Bayesian, multilevel, mixed-effect, logistic, regression model were used to analyze binary outcome variables any skin problem (presence or absence of any one of three skin conditions such as scabies, tungiasis, and tinea) and presence or absence of scabies, tungiasis, and tinea.

### Multilevel, mixed-effect, logistic regression (Frequentist approach)

We nested our data at three levels: individuals (level 1) within classrooms (level 2) within schools (level 3). Clustering effects at the school and classroom levels, including potential confounders and effect modifications, were assessed using multivariate, multilevel, mixed-effect, logistic regression and stratified analysis. Independent variables were checked for collinearity before the multivariate analysis. In both bivariate and multivariate analyses, we employed the model without random effects and with random school and class effects for all factors.

The null model contained no covariate variables and indicated whether to consider the random-effect model. Independent variables for which P < 0.25 in the bivariate, multilevel, mixed-effect, logistic regression model were included in all models, including the final model. To control for confounding, covariate variables for which P > 0.25 in the bivariate model were retained in the full multivariate model containing individual-, household- and school-level factors. These covariate variables included age in the model for any skin problem; body washing and household wealth in the scabies model; age, unclean fingernails, walking barefoot, family size, and access to health education on personal hygiene in the tungiasis model; and age, family size, and access to health education on personal hygiene in the tinea model.

The final model for any skin problem included sex; age; presence of unclean fingernails; presence of footwear; frequency of washing with soap; sharing beds, clothes, and combs; household wealth; and access to health education on personal hygiene. The scabies model included variables for sex; age; frequency of washing with soap; sharing beds, clothes, and combs; family size; household wealth; and access to health education on personal hygiene. The tungiasis model included variables for sex; age; nail trimming; walking barefoot; presence of footwear; frequency of washing with soap; sharing beds and clothes; family size; household wealth; and access to health education on personal hygiene. The tinea model included sex; age; presence of unclean fingernails; walking barefoot; presence of footwear; frequency of washing with soap; sharing beds and clothes; family size; household wealth; and access to health education on personal hygiene. Intra-cluster correlation coefficients (ICCs) for clustering effects at the school and classroom levels were reported for each model. We calculated the Akaike information criterion to evaluate model performance, with lower values indicating better fit [47].

### Bayesian multilevel, mixed-effect, logistic regression

The Bayesian method provides probabilistic summaries for the parameters of interest, treating all model parameters as random quantities. Unlike the frequentist model, Bayesian analysis uses prior knowledge and provides interval estimates (credible intervals) in which the value of the unknown parameter lies [48]. We used a multilevel approach, assuming that the risks of skin problems across schools and classrooms were correlated. Independent variables where P < 0.25 in the bivariate, multilevel, mixed-effect, logistic regression model were used to estimate skin problems in the Bayesian model.

We estimated model parameters using Markov Chain Monte Carlo simulation. Primarily, we used uniform priors for slopes, normal prior for the intercept, and inverse-gamma prior for the variance parameter for all models. In addition, we formulated an informative prior using previous related studies, for scabies [11, 30–32, 49], tungiasis [16, 19], and tinea [16, 22]. We then considered informative priors for slopes, normal prior for the intercept, and inverse-gamma prior for the variance parameter for these three models. However, we didn’t find compiled report of scabies, tungiasis, and tinea infection to formulate informative prior for any skin problem from previous studies. Thus, we used uniform priors for slopes, normal prior for the intercept, and inverse-gamma prior for the variance parameter. We ran 25,000 interations in the simulation, discarding the first 5,000, withdrawing 20,000 samples after values were thinned by 10, and storing values to compute the posterior probability. Before drawing samples, we tested parameters for Monte Carlo standard errors, and uncertainty due to simulation errors was confirmed to be below 5% of the standard deviation [35, 50].

An acceptance rate below 10% was used to identify a convergence problem in the Markov Chain Monte Carlo methods. For any skin problem, scabies, tungiasis, and tinea in the Bayesian models, the value of the acceptance rate according to the Metropolis-Hasting algorithm was 37% out of 20,000 proposed parameters. Efficiencies of at least 10% were considered good, and average efficiencies of 26% were achieved for any skin problem, 18% for scabies, 23% for tungiasis, and 24% for tinea. Convergence also was checked using diagnostic plots for all parameters. In the posterior estimate, the mean, SD, Monte Carlo standard error, median, credible intervals, and variance of the random effect were estimated for all parameters. ICCs measuring the clustering effects at the school and classroom levels were reported for each model. We did not find a postestimation command in STATA for the ICC calculation in the Bayesian approach. Therefore, we calculated the ICC using the following formulas:

$$ICC\;at\;school‐level=\frac{School\;level\;variance\;}{School+Class\;level\;variance+\frac{\pi 2}{3}},\;and$$

$$ICC\;at\;class‐level=\frac{School+Class\;level\;variance}{School+Class\;level\;variance+\pi 2/3},$$

where π^2^/3 = 3.29 indicates variance at the individual level in the logistic distribution [51, 52]. We used the deviance information criterion to evaluate model performance, with lower values indicating better fit.

## Results

The 861 schoolchildren (483 boys and 378 girls) in this study were aged 7 to 14 years, with a mean [SD] age of 11.4 [1.9] years. All recruited children’s parents or guardians participated in the household survey. The family size ranged from 3 to 14 (mean 6.7) persons. Among parents, 88.4% (761/861) of mothers and 48.8% (420/861) of fathers had never attended school. More than half of the mothers (54%; 463/857) were housewives, and most fathers (77.4%; 619/800) were farmers. One third of the children (33.3%; 287/861) lived in a poor household (S1 Table).

### Children’s personal hygiene and school hygiene

At the time of the interview, most children 81.9% (705/861) had trimmed fingernails, 24.3% (209/861) had unclean fingernails, and 2.7% (23/861) habitually walked barefoot. More than half washed their body 57.1% (492/861) and hair 54.1% (466/861) with soap every week. About 47.3% (407/861) washed their legs and feet with soap once daily. Almost two thirds shared beds (65.6%; 565/861), clothes (40.2%; 346/861), and combs (72%; 620/861) with other family members (S2 Table). Among schools, none had access to drinking water or handwashing facilities. All schools in this study had pit latrines covered with cement, but latrine floors were unclean, and no toilet paper was available (S1 Data).

### Prevalence of scabies, tungiasis, and tinea infections

As shown in Table 1, of 861 children examined for skin problems, 71.7% (617/861 [95% CI 68.6, 74.6]) had scabies, tungiasis, or tinea infections. Of those, 60% (370/617) were boys and 40% (247/617) girls. We found scabies in 5.3% (46/861 [95% CI 4.0, 7.1]) of children, all of whom had lesions in the interdigital places, hands, wrists, arms, and elbows, and 84.8% (39/46) of whom had lesions on the abdomen, chest, mamilla, or perimammillar area. The prevalence of tungiasis was 54.4% (468/861 [95% CI 51.0, 57.7]), 93.2% (436/468) of whom had infection on the feet and 6.8% (32/468) on both feet and hands. Of the 861 children, 39.1% (337/861 [95% CI 35.9, 42.5]) had tinea infections, most commonly tinea capitis (65%; 219/337). S3 Table presents the proportions of children with scabies, tungiasis, and tinea infections in relation to individual, household, and school factors.

**Table 1:** Distribution of scabies, tungiasis, and tinea infections among schoolchildren in the Wonago district, southern Ethiopia, 2017.

Skin problem	N	n	Frequency	Percentage
**Scabies**	861		46	5.3
Hands, interdigital places, wrists, arms, elbows		46	46	100
Armpit		46	22	47.8
Abdomen, chest, mamilla, perimammillar area		46	39	84.8
Back area, buttock and genitals		46	35	76.1
Feet area		46	34	73.9
**Tungiasis**	861		468	54.4
Feet		468	436	93.2
Feet and hands		468	32	6.8
**Tinea**	861		337	39.1
Tinea capitis (scalp)		337	219	65.0
Tinea corporis (body)		337	104	30.9
Tinea pedis (foot)		337	90	26.7
Tinea unguium (toenails or fingernails)		337	83	24.6
Tinea cruris (groin)		337	22	6.5
Tinea manuum (hands)		337	12	3.6
Tinea faciei (face)		337	8	2.4

N: Total number of children examined; n: children with skin problems

### Multilevel, mixed-effect, logistic regression (Frequentist and Bayesian approach)

S4, S5, S6, and S7 Tables show the frequentist approach for the full model. The clustering effect measured by ICC was 8.8% for prevalence of any of the three skin conditions, 2.8% for tungiasis, and 4.9% for tinea at the classroom level but low or insignificant at the school level, indicating that variability in prevalence was not attributable to school-level factors. The variability in scabies prevalence attributable to the classroom level was 28% and 8% at the school level. Low Akaike information criteria value were considered a better fit in the frequentist approach; for all outcomes, the full model containing individual-, household-, and school-level factors had low criteria values.

In the Bayesian model, the clustering effect measured by the ICC for any skin problem, scabies, tungiasis, or tinea was significant at both the school and classroom levels. The ICC values calculated in the Bayesian model were higher than those in the frequentist model. For instance, we observed 16% of classroom- and 5.3% of school-level variability for any skin problem (S8 Table); 49.3% of classroom- and 31.2% of school-level variability for scabies (S9 Table); 8.5% of classroom- and 3% of school-level variability for tungiasis (S10 Table); and 12.7% of classroom- and 5.7% of school-level variability for tinea (S11 Table). The full model containing individual- household-, and school-level factors showed a lower deviance information criterion value for any skin problems, scabies, tungiasis, and tinea models and thus was considered a better fit in the Bayesian approach.

### Risk factors for any skin problem

Using the frequentist model, the bivariate and multivariate analysis of any skin problem model revealed that sex; unclean fingernails; frequency of washing body, hair, legs, and feet with soap; sharing beds, clothes, and combs; and household wealth were predictive factors (Tables 2 and S4).

**Table 2:** Frequentist and Bayesian multivariate, multilevel, mixed-effect, logistic regression analysis of skin problem among schoolchildren in the Wonago district, southern Ethiopia, 2017.

Variables		Any skin problem				
				Frequentist (Maximum Likelihood Estimation)		Bayesian estimation
				Multilevel, mixed-effect, logistic regression		Bayesian multilevel, mixed-effect, logistic regression
		Yes (n (%)	No (n (%)	Adjusted odds ratio (95% CI)	P-value	Adjusted 95% BCI odds ratio (95% BCI)
**Individual child factors**						
Sex	Boys	370 (76.6)	113 (23.4)	1.52 (1.02, 2.27)	0.04	1.55 (1.01, 2.28)*
	Girls	247 (65.3)	131 (34.7)	1.0		1.0
Age in years	Mean (SD)			1.02 (0.90, 1.16)	0.74	1.02 (0.89, 1.15)
Unclean fingernails	Yes	165 (78.9)	44 (21.1)	1.72 (1.05, 2.82)	0.031	1.85 (1.08, 2.97)*
	No	452 (69.3)	200 (30.7)	1.0		1.0
Presence of footwear during examination	Yes	603 (72.0)	235 (28.0)	2.53 (0.82, 7.78)	0.105	2.68 (0.74, 6.91)
	No	14 (60.9)	9 (39.1)	1.0		1.0
Frequency of washing body with soap	Once per week	326 (66.3)	166 (33.7)	1.0		1.0
	Every two weeks	291 (78.9)	78 (21.1)	1.81 (1.20, 2.75)	0.005	1.90 (1.21, 2.85)*
Frequency of washing hair with soap	Once per week	284 (60.9)	182 (39.1)	1.0		1.0
	Every two weeks	333 (84.3)	62 (15.7)	2.92 (1.95, 4.39)	0.000	3.07 (1.98, 4.57)*
Frequency of washing legs and feet with soap	Once per day	222 (54.6)	185 (45.5)	0.23 (0.15, 0.35)	0.000	0.23 (0.15, 0.33)*
	Sometimes	395 (87.0)	59 (13.0)	1.0		1.0
Sharing beds	No	165 (55.7)	131 (44.3)	1.0		1.0
	Yes	452 (80.0)	113 (20.0)	1.92 (1.28, 2.87)	0.001	1.97 (1.27, 2.89)*
Sharing clothes	No	302 (58.6)	213 (41.4)	1.0		1.0
	Yes	315 (91.0)	31 (9.0)	5.13 (3.09, 8.50)	0.000	5.65 (3.31, 9.21)*
Sharing combs	No	115 (47.7)	126 (52.3)	1.0		1.0
	Yes	502 (81.0)	118 (19.0)	3.45 (2.23, 5.34)	0.000	3.65 (2.28, 5.53)*
**Household factors**						
Wealth status	Poor	223 (77.7)	64 (22.3)	1.71 (1.04, 2.79)	0.035	1.76 (1.03, 2.83)*
	Middle	210 (70.7)	87 (29.3)	1.12 (0.69, 1.83)	0.643	1.13 (0.66, 1.79)
	Rich	184 (66.4)	93 (33.6)	1.0		1.0
**School factors**						
Access to health education on personal hygiene	Yes	472 (70.0)	202 (30.0)	1.03 (0.54, 1.94)	0.932	1.26 (0.58, 2.44)
	No	145 (77.5)	42 (22.5)	1.0		1.0

BCI: Bayesian credible interval; CI: confidence interval; SD: standard deviation; *significant

As shown in Table 2, in the Bayesian multivariate analysis, boys had higher overall risk (aOR 1.55 [95% BCI 1.01, 2.28), as did children who had unclean fingernails (aOR 1.85 [95% BCI 1.08, 2.97]); who did not wash their body (aOR 1.90 [95% BCI 1.21, 2.85]) and hair (aOR 3.07 [95% BCI 1.98, 4.57]) with soap every week; who shared beds (aOR 1.97 [95% BCI 1.27, 2.89]), clothes (aOR 5.65 [95% BCI 3.31, 9.21]), or combs (aOR 3.65 [95% BCI 2.28, 5.53]); and who lived in poor households (aOR 1.76 [95% BCI 1.03, 2.83]). The odds were low among children who washed their legs and feet with soap every day (aOR 0.23 [95% BCI 0.15, 0.33]). S8 Table shows the results of the Bayesian model, including posterior mean, SD, Monte Carlo standard error, and median.

### Risk factors for scabies

Using a frequentist model in both the bivariate and multivariate analyses, the odds of scabies increased among boys (aOR 2.07 [95% CI 1.02, 4.23]) and children who shared beds (aOR 2.97 (95% CI 1.22, 7.21]) or combs (aOR 3.68 [95% CI 1.31, 10.3), as shown in S5 Table. Similarly, in the Bayesian multivariate analysis, the odds of scabies increased among boys (aOR 2.62 [95% BCI 1.19, 5.19]) and children who shared beds (aOR 4.30 [95% BCI 1.55, 10.4]) or combs (aOR 5.77 [95% BCI 1.72, 16.1]), as shown in S9 Table.

### Risk factors for tungiasis

As shown in S6 Table, using the frequentist model, the following variables were associated with tungiasis in both the bivariate and multivariate analyses: frequency of body, leg, and feet washing with soap; sharing beds or clothes; and household wealth status.

As shown in Table 3, the Bayesian multivariate analysis revealed that the odds of tungiasis increased among children who wore footwear at examination time (aOR 8.04 [95% BCI 2.46, 21.4]); who did not wash their body with soap every week (aOR 1.59 [95% BCI 1.13, 2.17]); who shared beds (aOR 2.00 [95% BCI 1.43, 2.71]) or clothes (aOR 2.85 [95% BCI 2.01, 3.97]); or who lived in poor households (aOR 1.93 [95% BCI 1.29, 2.79]). Rate were low among children who washed their legs and feet with soap every day (aOR 0.48 [95% BCI 0.35, 0.65]), compared with those who sometimes washed their legs and feet. S10 Table shows the results, including posterior mean, SD, Monte Carlo standard error, and median.

**Table 3:** Frequentist and Bayesian multivariate, multilevel, mixed-effect, logistic regression analysis of tungiasis among schoolchildren in the Wonago district, southern Ethiopia, 2017.

Variables		Tungiasis				
				Frequentist (Maximum Likelihood Estimation)		Bayesian estimation
				Multilevel, mixed-effect, logistic regression		Bayesian multilevel, mixed-effect, logistic regression
		Yes (n (%)	No (n (%)	Adjusted odds ratio (95% CI)	P-value	Adjusted 95% BCI odds ratio (95% BCI)
**Individual child factors**						
Sex	Boys	274 (56.7)	209 (43.3)	0.98 (0.72, 1.32)	0.890	1.08 (0.78, 1.46)
	Girls	194 (51.3)	184 (48.7)	1.0		1.0
Age in years	Mean (SD)			1.01 (0.92, 1.11)	0.790	1.01 (0.91, 1.11)
Fingernails trimmed	Yes	373 (52.9)	332 (47.1)	0.76 (0.51, 1.13)	0.178	0.93 (0.60, 1.38)
	No	95 (60.9)	61 (39.1)	1.0		1.0
Habit of walking barefoot	Always in barefoot	11 (50.0)	11 (50.0)	0.54 (0.21, 1.42)	0.211	2.41 (0.71,6.39)
	Sometimes in barefoot	225 (56.3)	175 (43.7)	1.13 (0.82, 1.56)	0.444	1.40 (0.99, 1.93)
	Never in barefoot	232 (52.8)	207 (47.2)	1.0		1.0
Presence of footwear during exam	Yes	459 (54.8)	379 (45.2)	2.07 (0.80, 5.37)	0.134	8.04 (2.46, 21.4)*
	No	9 (39.1)	14 (60.9)	1.0		1.0
Frequency of washing body with soap	Once per week	245 (49.8)	247 (50.2)	1.0		1.0
	Every two weeks	223 (60.4)	146 (39.6)	1.41 (1.03, 1.93)	0.031	1.59 (1.13, 2.17)*
Frequency of washing legs and feet with soap	Once per day	165 (40.5)	242 (59.5)	0.41 (0.30, 0.56)	0.000	0.48 (0.35, 0.65)*
	Sometimes	303 (66.7)	151 (33.3)	1.0		1.0
Sharing beds	No	121 (41.2)	174 (58.8)	1.0		1.0
	Yes	346 (61.2)	219 (38.8)	1.83 (1.34, 2.51)	0.000	2.00 (1.43, 2.71)*
Sharing clothes	No	230 (44.7)	285 (55.3)	1.0		1.0
	Yes	238 (68.8)	108 (31.2)	2.39 (1.72, 3.32)	0.000	2.85 (2.01, 3.97)*
**Household factors**						
Family size	1–4	45 (57.7)	33 (42.3)	1.0	1.0	
	≥5	423 (54.0)	360 (46.0)	0.80 (0.48, 1.35)	0.394	1.02 (0.58, 1.66)
Wealth status	Poor	174 (60.6)	113 (39.4)	1.51 (1.04, 2.20)	0.024	1.93 (1.29, 2.79)*
	Middle-class	158 (53.2)	139 (46.8)	1.08 (0.74, 1.58)	0.641	1.36 (0.89, 1.98)
	Rich	136 (49.1)	141 (50.9)	1.0		1.0
**School factors**						
Access to health education on personal hygiene	Yes	364 (54.0)	310 (46.0)	1.03 (0.68, 1.58)	0.834	1.52 (0.087, 2.54)
	No	104 (55.6)	83 (44.4)	1.0		1.0

BCI: Bayesian credible interval; CI: confidence interval; SD: standard deviation; *significant

### Risk factors for tinea infections

In both the bivariate and multivariate analyses (S7 Table), using the frequentist model, tinea was associated with sex; unclean fingernails; frequency of washing hair, legs, and feet with soap; sharing beds, clothes, or combs.

As indicated in Table 4, in the Bayesian multivariate analysis, the odds of tinea increased among boys (aOR 2.42 [95% BCI 1.72, 3.34]), children with unclean fingernails (aOR 2.96 [95% BCI 1.96, 4.31]), children who did not wash their hair with soap every week (aOR 1.94 [95% BCI 1.39, 2.66]), children who shared beds (aOR 1.78 [95% BCI 1.22, 2.51]), clothes (aOR 1.89 [95% BCI 1.30, 2.65]), or combs (aOR 2.93 [95% BCI 1.90, 4.37]). Rates were high among children who lived in households with more than or equal to five family size (aOR 2.25 [95% BCI 1.20, 3.92]); and children who lived in poor (aOR 1.99 [95% BCI 1.29, 2.94]) and middle-class (aOR 2.01 [95% BCI 1.30, 2.98]) households, compared to those who lived in rich households. S11 Table shows the results, including posterior mean, SD, Monte Carlo standard error, and median.

**Table 4:** Frequentist and Bayesian multivariate multilevel, mixed-effect, logistic regression analysis of tinea infections among schoolchildren in the Wonago district, southern Ethiopia, 2017.

Variables		Tinea infections				
				Frequentist (Maximum Likelihood Estimation)		Bayesian estimation
				Multilevel, mixed-effect, logistic regression		Bayesian multilevel, mixed-effect, logistic regression
		Yes (%)	No (%)	Adjusted odds ratio (95% CI)	P-value	Adjusted 95% BCI odds ratio (95% BCI)
**Individual child factors**						
Sex	Boys	226 (46.8)	257 (53.2)	1.92 (1.40, 2.63)	0.000	2.42 (1.72, 3.34)*
	Girls	111 (29.4)	267 (70.6)	1.0		1.0
Age in years	Mean (SD)			1.02 (0.92, 1.13)	0.697	1.05 (0.94, 1.16)
Unclean fingernails	Yes	106 (50.7)	103 (49.3)	2.02 (1.40, 2.92)	0.000	2.96 (1.96, 4.31)*
	No	231 (35.4)	421 (64.6)	1.0		1.0
Frequency of washing body with soap	Once per week	183 (37.2)	309 (62.8)	1.0		1.0
	Every two weeks	154 (41.7)	215 (58.3)	1.06 (0.76, 1.46)	0.741	1.36 (0.95, 1.88)
Frequency of washing hair with soap	Once per week	153 (32.8)	313 (67.2)	1.0		1.0
	Every two weeks	184 (46.6)	211 (53.4)	1.53 (1.13, 2.08)	0.006	1.94 (1.39, 2.66)*
Frequency of washing legs and feet with soap	Once per day	126 (31.0)	281 (69.0)	0.66 (0.48, 0.91)	0.011	0.89 (0.63, 1.23)
	Sometimes	211 (46.5)	243 (53.5)	1.0		1.0
Sharing beds	No	84 (28.4)	212 (71.6)	1.0		1.0
	Yes	253 (44.8)	312 (55.2)	1.48 (1.06, 2.08)	0.021	1.78 (1.22, 2.51)*
Sharing clothes	No	158 (30.7)	357 (69.3)	1.0		1.0
	Yes	179 (51.7)	167 (48.3)	1.63 (1.17, 2.27)	0.004	1.89 (1.30, 2.65)*
Sharing combs	No	62 (25.7)	179 (74.3)	1.0		1.0
	Yes	275 (44.4)	345 (55.6)	2.15 (1.46, 3.17)	0.000	2.93 (1.90, 4.37)*
**Household factors**						
Family size	1–4	27 (34.6)	51 (65.4)	1.0		1.0
	≥5	310 (39.6)	473 (60.4)	1.24 (0.73, 2.12)	0.428	2.25 (1.20, 3.92)*
Wealth status	Poor	119 (41.5)	168 (58.5)	1.19 (0.81, 1.75)	0.379	1.99 (1.29, 2.94)*
	Middle-class	120 (40.4)	177 (59.6)	1.23 (0.84, 1.81)	0.285	2.01 (1.30, 2.98)*
	Rich	98 (35.4)	179 (64.6)	1.0		1.0
**School factors**						
Access to health education on personal hygiene	Yes	253 (37.5)	421 (62.5)	0.92 (0.58, 1.45)	0.706	1.11 (0.62, 1.88)
	No	84 (44.9)	103 (55.1)	1.0		1.0

BCI: Bayesian credible interval; CI: confidence interval; SD: standard deviation; *significant

## Discussion

In a randomly selected, representative sample of schoolchildren aged 7 to 14 years in the Gedeo zone of southern Ethiopia, almost three-fourths had at least one type of skin problem, most commonly tungiasis and tinea infections. Unlike previous studies in Ethiopia, we examined the skin from the scalp to the toes and assessed children for scabies, tungiasis, and tinea. Using two approaches, the frequentist model and the Bayesian model, and adjusting for the effects of clustering at the school and classroom levels, we found associations between these skin problems and several variables (sex; fingernail condition; washing with soap; sharing beds, clothes, or combs; and household wealth). Unlike the frequentist model, a Bayesian model uses fixed data, and the estimated parameters are viewed as random. It also includes prior information and does not rely on sampling, instead using probability to indicate uncertainty in the model [34]. Interpreting credible intervals is easier in the Bayesian model than in the frequentist model because the range of values in the Bayesian approach has a direct probabilistic interpretation of the true estimate [34, 35]. In our model, we clustered individual data within the same classroom and classrooms within the same school. To account for clustering effects, we applied a multilevel analysis using both the frequentist and Bayesian approaches. Although both models yielded similar estimates, the Bayesian model had smaller standard errors compared with the frequentist model and thus demonstrated better performance [34]. The similarity in estimates of the two methods is probably due to the large sample size, particularly for the frequentist method. In the Bayesian Markov Chain Monte Carlo simulations, we applied a large number of iterations to achieve convergence. Moreover, compared with the frequentist approach, the Bayesian model demonstrated high clustering effects at the school and classroom levels, indicating the presence of shared risk factors for skin problems and underscoring the importance of the Bayesian approach for explaining the observed variations in skin problems.

The present study has some limitations. First, given its cross-sectional nature, causality between the outcome and exposure variables cannot be determined with certainty. Second, we diagnosed skin problems clinically but not via skin scrapings, as fungal cultures were not available in our setting [53]. Thus, the prevalence of tinea infections could be under or overestimated. However, our results align with those of other studies, including a recent study showing that almost 90% of clinically suspected tinea infections were confirmed by microscopy [54]. Some of the information related to personal hygiene may be prone to reporting biases, and observer bias may occur in recording the outcome variables. To address this issue, we had two examiners conduct exams on 10% of the children, which helped ensure reproducibility and agreement. The nonsignificant effect of habitually walking barefoot in the Bayesian model could be due to unmeasured confounding factors, such as type of footwear. Although we focused on scabies, tungiasis, and tinea infections and not other skin problems, we believe that our study provides relevant information regarding the epidemiology of skin conditions in general in the study area.

Climate variation may play an important role in the occurrence of skin problems [55–57], but we did not assess it in our cross-sectional survey. Further studies should investigate the role of climate in skin problems. The health-seeking behavior of parents for children with skin problems also needs to be assessed at the community level. We conducted our study using a large representative sample of rural schoolchildren. We estimated the parameters using randomly generated Markov Chain Monte Carlo samples using a Bayesian approach. Therefore, the simulated data are large and close to the real population, making the findings generalizable to school-aged children in similar socio-economic and cultural settings.

ICC values for nested data are rarely reported in previous studies [58]. We calculated the ICC values using both the frequentist and Bayesian models. Compared to the frequentist model, the Bayesian model yielded higher clustering effects for skin problems at the school and classroom levels, suggesting that the Bayesian approach better explains variations in skin problems. In particular, the observed high variation in scabies prevalence at the school and classroom levels suggests increased risk of scabies due to close contact in these locations. The observed clustering of tungiasis prevalence at the classroom level indicates a commonality in the classroom (e.g., earthen or dusty or cracked floor) that could result in clustering.

Our finding of a 5% prevalence for scabies aligns with that of similar studies in southern Ethiopia [16] and with the 6% reported in the national survey among schoolchildren [49], though these findings are much lower than previous reports of 34% to 50% in the same region [11, 32]. These variations could be due to differences in personal hygiene, socioeconomic factors, study settings, climate variations, and disease outbreaks. Scabies varied with seasons [56, 57, 59]. Our study period was in dry season, this could also affect scabies prevalence because scabies is more common in cool and humid weather condition [57, 59]. In contrast, other evidence indicates that an increase in temperature, is associated with increased scabies prevalence [56]. It has previously been reported that the highest cases of scabies in community based study in drought affected area in Ethiopia [30]. Though we did not assess the seasonal variations in this study due to the nature of our study design, it might be linked with overcrowding and close contact in the cooler seasons. Other studies, however, did not find significant variations with seasons [7, 60, 61]. The prevalence of tungiasis was 54%, which aligns with other findings from rural southern Ethiopia [19] and Kenya [62] but exceeds the 35% found in other areas in southern Ethiopia [16]. The discrepancy may be explained by our rural study setting, where most children live in houses with earthen floors and attend classrooms with poor hygiene facilities. It also could be linked with seasonal variations because our study period was in dry season, where tungiasis is more common [2, 63, 64]. This may be explained with indirect link with access to water in the dry season for personal hygiene protection. The prevalence of tinea infections was 39%, which aligns with rates in other rural areas of southern Ethiopia [22] but exceeds those from urban areas of Ethiopia [16], from Cote d’Ivoire [65], and from Tanzania [66]. The variations could be associated with personal hygiene, poverty, and climate conditions [55], as well as immune factors [67–69]. Consistent with findings elsewhere in Ethiopia [22, 29, 70] and other African studies [65, 71–73], boys were more likely to be affected by tinea infection (particularly tinea capitis) than girls, perhaps due to the higher risk associated with frequent shaving or haircuts [55] or due to personal hygiene (e.g., frequency of hair washing).

Nail hygiene is a well-known factor preventing the spread of infection. Many pathogens can be shed from fingernails, resulting in transmission [74]. We found that children with unclean fingernails had higher risks of skin problems, particularly tinea. Infrequent body and hair washing with soap also is associated with increased skin problems [31, 62, 75, 76]. Our results similarly show that washing the feet daily with soap was a protective factor of both, tungiasis and tinea. Sharing beds, clothes, or combs also was associated with increased risk of scabies and tinea infections, as documented by other studies in Ethiopia [11, 32] and other African countries [75–78].

Similar to previous reports from Ethiopia [19] and Rwanda [15], we observed no differences in tungiasis rates among boys and girls. Our bivariate frequentist model identified boys as slightly more at risk (p = 0.103), perhaps due to behavioral factors related to hygiene. Findings by Elson et al. [62] and Wiese et al. [79] also show higher risk of tungiasis among boys. Other evidence suggests that the risk of acquiring tungiasis varies with age-specific behavioral patterns [62, 79] and increases among children younger than 15 years [62] and the elderly [79]. We could not confirm these findings as our study did not include adolescents older than 14 years or older age groups. Previous reports indicate that poor hygiene, including low frequency of washing with soap [79], is associated with increased tungiasis rates [15, 80]. We similarly found that children who did not wash with soap every week were more likely to be affected by tungiasis. Transmission also is more likely in places where shoes are not worn, such as sleeping rooms [81]. Our findings confirmed that sharing beds and clothes increased risk of tungiasis.

Poverty is an important risk factor for skin problems [82]. In our study, children living in poor households were more likely to be affected by skin problems, perhaps because these families cannot afford soap and other hygiene products. Housing construction material [83], contact with domestic animals [84, 85], and poor environmental hygiene [80, 81] also could be contributing factors. Studies from Kenya [62, 83] and Nigeria [86] show that floor type plays an important role in tungiasis transmission. Although we did not conduct a separate analysis for floor material in the multivariate model, we included it as part of our wealth index construction. We observed a high prevalence of tungiasis among children living in houses constructed with natural earth or dung floors. Dry, loose soil or sand is a favorable environment for tungiasis-transmitting fleas [62, 86]. Evidence suggests that large family size, is associated with the risk of acquiring skin problems [87]. We found that children who lived in households of large family size had higher risks of skin problems, particularly tinea infections, probably due to close contact as a result of overcrowding.

Foot hygiene and appropriate footwear provide important protection [88] against tinea [89, 90] and tungiasis [86, 91]. Walking barefoot is a known risk factor for tungiasis [15, 19]. In this study, walking barefoot was not associated with skin problems in either the Bayesian or frequentist model. The nonsignificant effect in the Bayesian model may be due to other unmeasured factors, such as type of footwear. However, the association between presence of foot wear during examination time and an increased risk of tungiasis was unexpected and should be interpreted with caution. A previous study has shown the association between wearing closed footwear and an increased risk of tungiasis [16].

## Conclusion

Skin problems such as scabies, tungiasis, and tinea infections are major health concerns among schoolchildren in rural Ethiopia. National school health programs aim to detect active cases and apply preventive measures via periodic screenings, but most of these programs are non-functional due to lack of funding or resources [23]. We found significant associations between many individual hygiene-related factors and scabies, tungiasis and tinea. In particular, our findings highlight the link between skin problems and poverty.

Health education and regular checkups by schoolteachers can be vital to improving rates of infection. Education should emphasize the importance of regular washing of legs and feet with soap and appropriate footwear. The observed high clustering effects for skin problems at the school and classroom levels indicate that transmission occurs and may be more likely in these environments, probably via close contact. Important measures to prevent transmission include access to adequate water and soap in all schools, and reduced class sizes. Contagious and highly transmissible skin problems are important policy issues in Ethiopia. Addressing these issues will require efforts from the Ministry of Health and Education to reduce major gaps in school health services implementation and to improve children’s health, particularly among rural schoolchildren in areas such as Gedeo.

## Supporting information

S1 Checklist STROBE checklist. (DOCX)

S1 Fig Flowchart of study inclusion. (TIF)

S1 Data Dataset. (XLSX)

S1 Table Demographic and socio-economic characteristics of schoolchildren and their parents in the Wonago district of southern Ethiopia, 2017. (DOCX)

S2 Table Personal hygiene of schoolchildren in the Wonago district, southern Ethiopia, 2017. (DOCX)

S3 Table The prevalence of skin problems in relation to individual, household and school factors among schoolchildren in the Wonago district, southern Ethiopia, 2017. (DOCX)

S4 Table Bivariate and multivariate, multilevel, mixed-effect, logistic regression analysis of skin problem among schoolchildren in the Wonago district, southern Ethiopia, 2017. (DOCX)

S5 Table Bivariate and multivariate, multilevel, mixed-effect, logistic regression analysis of scabies among schoolchildren in the Wonago district, southern Ethiopia, 2017. (DOCX)

S6 Table Bivariate and multivariate, multilevel, mixed-effect, logistic regression analysis of tungiasis among schoolchildren in the Wonago district, southern Ethiopia, 2017. (DOCX)

S7 Table Bivariate and multivariate multilevel, mixed-effect, logistic regression analysis of tinea infections among schoolchildren in the Wonago district, southern Ethiopia, 2017. (DOCX)

S8 Table Bayesian multivariate, multilevel, mixed-effect, logistic regression analysis of skin problem among schoolchildren in the Wonago district, southern Ethiopia, 2017. (DOCX)

S9 Table Bayesian multivariate, multilevel, mixed-effect, logistic regression analysis of scabies among schoolchildren in the Wonago district, southern Ethiopia, 2017. (DOCX)

S10 Table Bayesian multivariate, multilevel, mixed-effect, logistic regression analysis of tungiasis among schoolchildren in the Wonago district, southern Ethiopia, 2017. (DOCX)

S11 Table Bayesian multivariate, multilevel, mixed-effect, logistic regression analysis of tinea infections among schoolchildren in the Wonago district, southern Ethiopia, 2017. (DOCX)
