# Supplementary material for: Risk factors for scabies, tungiasis, and tinea infections among schoolchildren in southern Ethiopia: A cross-sectional Bayesian multilevel model
Source: PLoS Negl Trop Dis. 2021 Oct 6;15(10):e0009816. doi: 10.1371/journal.pntd.0009816 (PMC8494366; doi:10.1371/journal.pntd.0009816)
Supplement: S1 Checklist — (DOCX) [file pntd.0009816.s001.docx]

**S1 Checklist. STROBE checklist**

|  | **Item**  **number** | **Recommendation** | Manuscript section and line number |
| --- | --- | --- | --- |
| Title and Abstract | 1 | (a) Indicate the study's design with a commonly used term in the title or the abstract | Title |
|  |  | (b) Provide in the abstract an informative and balanced summary of what was done and what was found | Abstract: pages:2 and 3 |
| Introduction | | |  |
| Background/rationale | 2 | Explain the scientific background and rationale for the investigation being reported | Pages: 3 to 5 |
| Objectives | 3 | State specific objectives, including any prespecified hypotheses | Page: 4: lines; 63 to 65 |
| Methods | | |  |
| Study design | 4 | Present key elements of study design early in the paper | Page: 6: line:119 |
| Setting | 5 | Describe the setting, locations, and relevant dates, including periods of recruitment, exposure, follow-up, and data collection | Page: 6: lines: 117 to 119 |
| Participants | 6 | (a) Cohort study- Give the eligibility criteria, and the sources and methods of selection of participants. Describe methods of follow-up |  |
|  |  | Case-control study- Give the eligibility criteria, and the sources and methods of case ascertainment and control selection. Give the rationale for the choice of cases and controls |  |
|  |  | Cross-sectional study- Give the eligibility criteria, and the sources and methods of selection of participants | Pages: 6: 119 to 126  Page: 6: S1 Fig. |
|  |  | (b) Cohort study-For matched studies, give matching criteria and number of exposed and unexposed |  |
|  |  | Case-control study-For matched studies, give matching criteria and the number of controls per case |  |
| Variables | 7 | Clearly define all outcomes, exposures, predictors, potential confounders, and effect modifiers. Give diagnostic criteria, if applicable | Methods: lines: 136 to 144 |
| Data sources/ measurement | 8^a^ | For each variable of interest, give sources of data and details of methods of assessment (measurement). | Methods: lines: 136 to 144  Data collection tools and procedures; Lines: 128 to 144  Diagnosis and measurement of skin problems; line 145 to 175 |
|  |  | Describe comparability of assessment methods if there is more than one group |  |
| Bias | 9 | Describe any efforts to address potential sources of bias | Methods: Lines: 132 to 135 |
| Study size | 10 | Explain how the study size was arrived at | Methods: Study location, design, participants, and sample size: page 6: lines:120 to 126 |
| Quantitative variables | 11 | Explain how quantitative variables were handled in the analyses. If applicable, describe which groupings were chosen, and why | Methods: Statistical analysis: lines: 180 to 182 |
| Statistical methods | 12 | (a) Describe all statistical methods, including those used to control for confounding | Methods: Statistical analysis: Lines: lines: 176 to 257 |
|  |  | (b) Describe any methods used to examine subgroups and interactions |  |
|  |  | (c) Explain how missing data were addressed |  |
|  |  | (d) Cohort study-If applicable, explain how loss to follow-up was addressed |  |
|  |  | Cross-sectional study-If applicable, describe analytical methods taking account of sampling strategy | Methods: Statistical analysis: lines 186 to 187 |
|  |  | (e) Describe any sensitivity analyses |  |
| Results | | |  |
| Participants | 13^a^ | (a) Report the numbers of individuals at each stage of the study e.g., numbers potentially eligible, examined for eligibility, confirmed eligible, included in the study, completing follow-up, and analysed | Methods: S1 Fig. |
|  |  | (b) Give reasons for non-participation at each stage | Methods: S1 Fig. |
|  |  | (c) Consider use of a flow diagram | Methods: S1 Fig. |
| Descriptive data | 14^a^ | (a) Give characteristics of study participants (e.g., demographic, clinical, social) and information on exposures and potential confounders | Results: 259 to 265 |
|  |  | (b) Indicate the number of participants with missing data for each variable of interest |  |
|  |  | (c) Cohort study-Summarise follow-up time (e.g., average and total amount) |  |
| Outcome data | 15^a^ | Cohort study-Report numbers of outcome events or summary measures over time |  |
|  |  | Case-control study-Report numbers in each exposure category, or summary measures of exposure |  |
|  |  | Cross-sectional study-Report numbers of outcome events or summary measures | Results: 275 to 285 and Table.1 |
| Main results | 16 | (a) Give unadjusted estimates and, if applicable, confounder-adjusted estimates and their precision (e.g., 95% confidence interval). Make clear which confounders were adjusted for and why they were included | Results: Supporting information: Table S4, S5, S6, and S7  Table 2, 3,4 |
|  |  | (b) Report category boundaries when continuous variables were categorized | Result: S3 Table |
|  |  | (c) If relevant, consider translating estimates of relative risk into absolute risk for a meaningful time period | NA |
| Other analysis | 17 | Report other analyses done- e.g., analyses of subgroups and interactions, and sensitivity analyses |  |
| Discussion | | |  |
| Key results | 18 | Summarise key results with reference to study objectives | Discussion: Lines: 372 to 379 |
| Limitations | 19 | Discuss limitations of the study, taking into account sources of potential bias or imprecision.  Discuss both direction and magnitude of any potential bias. | Discussion: Lines: 396 to 417 |
| Interpretation | 20 | Give a cautious overall interpretation of results considering objectives, limitations, multiplicity of analyses, results from similar studies, and other relevant evidence. | Discussion: Lines: 418 to 494 |
| Generalisability | 21 | Discuss the generalisability (external validity) of the study results | Discussion: Lines: 415 to 417 |
| Other information | | |  |
| Funding | 22 | Give the source of funding and the role of the funders for the present study and, if applicable, for the original study on which the present article is based | The Norwegian Programme for Capacity Development in Higher Education and Research for Development-South Ethiopia Network Universities in Public Health project provided funding. |

^a^ Give such information separately for cases and controls in case-control studies, and, if applicable, for exposed and unexposed groups in cohort and cross-sectional studies.
