# Supplementary material for: Risk factors for scabies, tungiasis, and tinea infections among schoolchildren in southern Ethiopia: A cross-sectional Bayesian multilevel model
Source: PLoS Negl Trop Dis. 2021 Oct 6;15(10):e0009816. doi: 10.1371/journal.pntd.0009816 (PMC8494366; doi:10.1371/journal.pntd.0009816)
Supplement: S1 Table — (DOCX) [file pntd.0009816.s004.docx]

**S1 Table. Demographic and socio-economic characteristics of schoolchildren and their parents in the Wonago district of southern Ethiopia, 2017**

| **Variables** | | **Frequency** | **Percent** |
| --- | --- | --- | --- |
| Sex | Boys | 483 | 56.1 |
|  | Girls | 378 | 43.9 |
| Child age group | 7-9 | 158 | 18.4 |
|  | 10-14 | 703 | 81.6 |
| Family size | 1-4 | 78 | 9.1 |
|  | ≥5 | 783 | 90.9 |
| Mother’s education | No formal education | 761 | 88.4 |
|  | Primary | 83 | 9.6 |
|  | Secondary and above | 13 | 1.5 |
|  | Mother or guardian not alive | 4 | 0.5 |
| Mother’s occupation | Government employee | 8 | 0.9 |
|  | Farmer | 287 | 33.5 |
|  | Trader | 61 | 7.1 |
|  | Daily labourer | 32 | 3.7 |
|  | Housewife | 463 | 54.0 |
|  | Student | 6 | 0.7 |
| Father’s education | No formal education | 420 | 48.8 |
|  | Primary | 294 | 34.1 |
|  | Secondary and above | 86 | 10 |
|  | Father or guardian not alive | 61 | 7.1 |
| Father’s occupation | Government employee | 44 | 5.5 |
|  | Farmer | 619 | 77.4 |
|  | Trader | 126 | 15.7 |
|  | Daily labourer | 11 | 1.4 |
| Wealth status | Poor | 287 | 33.3 |
|  | Middle-class | 297 | 34.5 |
|  | Rich | 277 | 32.2 |
