# Supplementary material for: Risk factors for scabies, tungiasis, and tinea infections among schoolchildren in southern Ethiopia: A cross-sectional Bayesian multilevel model
Source: PLoS Negl Trop Dis. 2021 Oct 6;15(10):e0009816. doi: 10.1371/journal.pntd.0009816 (PMC8494366; doi:10.1371/journal.pntd.0009816)
Supplement: S2 Table — (DOCX) [file pntd.0009816.s005.docx]

S2 Table. Personal hygiene of schoolchildren in the Wonago district, southern Ethiopia, 2017

| **Variables** | | **Frequency** | **Percentage** |
| --- | --- | --- | --- |
| Finger nails trimmed | Yes | 705 | 81.9 |
|  | No | 156 | 18.1 |
| Unclean fingernails | Yes | 209 | 24.3 |
|  | No | 652 | 75.7 |
| Presence of footwear during examination | Yes | 838 | 97.3 |
|  | No | 23 | 2.7 |
| Frequency of washing body with soap | Once per week | 492 | 57.1 |
|  | Every two weeks | 369 | 42.9 |
| Frequency of washing hair with soap | Once per week | 466 | 54.1 |
|  | Every two weeks | 395 | 45.9 |
| Frequency of washing legs and feet with soap | Once per day | 407 | 47.3 |
|  | Sometimes | 454 | 52.7 |
| Sharing beds | No | 296 | 34.4 |
|  | Yes | 565 | 65.6 |
| Sharing clothes | No | 515 | 59.8 |
|  | Yes | 346 | 40.2 |
| Sharing combs | No | 241 | 28.0 |
|  | Yes | 620 | 72.0 |
