# Supplementary material for: Risk factors for scabies, tungiasis, and tinea infections among schoolchildren in southern Ethiopia: A cross-sectional Bayesian multilevel model
Source: PLoS Negl Trop Dis. 2021 Oct 6;15(10):e0009816. doi: 10.1371/journal.pntd.0009816 (PMC8494366; doi:10.1371/journal.pntd.0009816)
Supplement: S3 Table — (DOCX) [file pntd.0009816.s006.docx]

S3 Table. The prevalence of skin problems in relation to individual, household and school factors among schoolchildren in the Wonago district, southern Ethiopia, 2017

| **Variables** | | **Scabies** | | **Tungiasis** | | **Tinea infections** | |
| --- | --- | --- | --- | --- | --- | --- | --- |
| **Individual child factors** | | **Yes (n (%)** | **No (n (%)** | **Yes (n (%)** | **No (n (%)** | **Yes (n (%)** | **No (n (%)** |
| Sex | Boys | 31 (6.4) | 452 (93.6) | 274 (56.7) | 209 (43.3) | 226 (46.8) | 257 (53.2) |
|  | Girls | 15 (4.0) | 363 (95.0) | 194 (51.3) | 184 (48.7) | 111 (29.4) | 267 (70.6) |
| Age in years | 7-9 | 18 (11.4) | 140 (88.6) | 83 (52.5) | 75 (47.5) | 61 (38.6) | 97 (61.4) |
|  | 10-14 | 28 (4.0) | 675 (96.0) | 385 (54.8) | 318 (45.2) | 276 (39.3) | 427 (60.7) |
| Finger nails trimmed | Yes | 39 (5.5) | 666 (94.5) | 373 (52.9) | 332 (47.1) | 264 (37.5) | 441 (62.6) |
|  | No | 7(4.5) | 149 (95.5) | 95 (60.9) | 61 (39.1) | 73 (46.8) | 83 (53.2) |
| Unclean fingernails | Yes | 16 (7.7) | 193 (92.3) | 121 (57.9) | 88 (42.1) | 106 (50.7) | 103 (49.3) |
|  | No | 30 (4.6) | 622 (95.4) | 347 (53.2) | 305 (46.8) | 231 (35.4) | 421 (64.6) |
| Habit of walking barefoot | Always in barefoot | 0 | 22 (100) | 11 (50.0) | 11 (50.0) | 9 (40.9) | 13 (59.1) |
|  | Sometimes in barefoot | 24 (6.0) | 376 (94.0) | 225 (56.3) | 175 (43.7) | 157 (39.3) | 243 (60.7) |
|  | Never in barefoot | 22 (5.0) | 417 (95.0) | 232 (52.8) | 207 (47.2) | 171 (38.9) | 268 (61.1) |
| Presence of footwear during exam | Yes | 46 (5.5) | 792 (94.5) | 459 (54.8) | 379 (45.2) | 329 (39.3) | 509 (60.7) |
|  | No | 0 | 23 (100) | 9 (39.1) | 14 (60.9) | 8 (34.8) | 15 (65.2) |
| Frequency of washing body with soap | Once per week | 24 (4.9) | 468 (95.1) | 245 (49.8) | 247 (50.2) | 183 (37.2) | 309 (62.8) |
|  | Every two weeks | 22 (6.0) | 347 (94.0) | 223 (60.4) | 146 (39.6) | 154 (41.7) | 215 (58.3) |
| Frequency of washing hair with soap | Once per week | 20 (4.3) | 446 (95.7) | 218 (46.8) | 248 (53.2) | 153 (32.8) | 313 (67.2) |
|  | Every two weeks | 26 (6.6) | 369 (93.4) | 250 (63.3) | 145 (36.7) | 184 (46.6) | 211 (53.4) |
| Frequency of washing legs and feet with soap | Once per day | 16 (3.9) | 391 (96.1) | 165 (40.5) | 242 (59.5) | 126 (31.0) | 281 (69.0) |
|  | Sometimes | 30 (6.6) | 424 (93.4) | 303 (66.7) | 151 (33.3) | 211 (46.5) | 243 (53.5) |
| Sharing beds | No | 7 (2.4) | 289 (97.6) | 122 (41.2) | 174 (58.8) | 84 (28.4) | 212 (71.6) |
|  | Yes | 39 (6.9) | 526 (93.1) | 346 (61.2) | 219 (38.8) | 253 (44.8) | 312 (55.2) |
| Sharing clothes | No | 22 (4.3) | 493 (95.7) | 230 (44.7) | 285 (55.3) | 158 (30.7) | 357 (69.3) |
|  | Yes | 24 (6.9) | 322 (93.1) | 238 (68.8) | 108 (31.2) | 179 (51.7) | 167 (48.3) |
| Sharing combs | No | 5 (2.1) | 236 (97.9) | 96 (39.8) | 145 (60.2) | 62 (25.7) | 179 (74.3) |
|  | Yes | 41 (6.6) | 579 (93.4) | 372 (60.0) | 248 (40.0) | 275 (44.4) | 345 (55.6) |
| **Household factors** | |  |  |  |  |  |  |
| Family size | 1–4 | 0 | 78 (100) | 45 (57.7) | 33 (42.3) | 27 (34.6) | 51 (65.4) |
|  | ≥5 | 46 (5.9) | 737 (94.1) | 423 (54.0) | 360 (46.0) | 310 (39.6) | 473 (60.4) |
| Wealth status | Poor | 16 (5.6) | 271 (94.4) | 174 (60.6) | 113 (39.4) | 119 (41.5) | 168 (58.5) |
|  | Middle-class | 18 (6.1) | 279 (93.9) | 158 (53.2) | 139 (46.8) | 120 (40.4) | 177 (59.6) |
|  | Rich | 12 (4.3) | 265 (95.7) | 136 (49.1) | 141 (50.9) | 98 (35.4) | 179 (64.6) |
| **School factors** | |  |  |  |  |  |  |
| Access to health education on personal hygiene | Yes | 26 (3.9) | 648 (96.1) | 364 (54.0) | 310 (46.0) | 253 (37.5) | 421 (62.5) |
|  | No | 20 (10.7) | 167 (89.3) | 104 (55.6) | 83 (44.4) | 84 (44.9) | 103 (55.1) |
