# Supplementary material for: Risk factors for scabies, tungiasis, and tinea infections among schoolchildren in southern Ethiopia: A cross-sectional Bayesian multilevel model
Source: PLoS Negl Trop Dis. 2021 Oct 6;15(10):e0009816. doi: 10.1371/journal.pntd.0009816 (PMC8494366; doi:10.1371/journal.pntd.0009816)
Supplement: S4 Table — (DOCX) [file pntd.0009816.s007.docx]

**S4 Table. Bivariate and multivariate, multilevel, mixed-effect, logistic regression analysis of skin problem among schoolchildren in the Wonago district, southern Ethiopia, 2017**

| **Variables** | | **Any skin problem** | | | | | |
| --- | --- | --- | --- | --- | --- | --- | --- |
| **Individual child factors** | | **Yes (n (%)** | **No (n (%)** | **Crude odds ratio (COR) (95% CI)** | **P-value** | **Adjusted OR (95% CI)** | **P-value** |
| Sex | Boys | 370 (76.6) | 113 (23.4) | 1.73 (1.27, 2.36) | 0.000 | 1.52 (1.02, 2.27) | 0.042 |
|  | Girls | 247 (65.3) | 131 (34.7) | 1.0 |  | 1.0 |  |
| Age in years (continuous) | Mean (SD) |  |  | 0.95 (0.86, 1.05) | 0.290 | 1.02 (0.90, 1.16) | 0.740 |
| Unclean fingernails | Yes | 165 (78.9) | 44 (21.1) | 1.69 (1.14, 2.49) | 0.009 | 1.72 (1.05, 2.82) | 0.031 |
|  | No | 452 (69.3) | 200 (30.7) | 1.0 |  | 1.0 |  |
| Presence of footwear during exam | Yes | 603 (72.0) | 235 (28.0) | 2.13 (0.87, 5.24) | 0.099 | 2.53 (0.82, 7.78) | 0.105 |
|  | No | 14 (60.9) | 9 (39.1) | 1.0 |  | 1.0 |  |
| Frequency of washing body with soap | Once per week | 326 (66.3) | 166 (33.7) | 1.0 |  | 1.0 |  |
|  | Every two weeks | 291 (78.9) | 78 (21.1) | 2.11 (1.51, 2.94) | 0.000 | 1.81 (1.20, 2.75) | 0.005 |
| Frequency of washing hair with soap | Once per week | 284 (60.9) | 182 (39.1) | 1.0 |  | 1.0 |  |
|  | Every two weeks | 333 (84.3) | 62 (15.7) | 3.52 (2.51, 4.93) | 0.000 | 2.92 (1.95, 4.39) | 0.000 |
| Frequency of washing legs and feet with soap | Once per day | 222 (54.6) | 185 (45.5) | 0.17 (0.12, 0.24) | 0.000 | 0.23 (0.15, 0.35) | 0.000 |
|  | Sometimes | 395 (87.0) | 59 (13.0) | 1.0 |  | 1.0 |  |
| Sharing beds | No | 165 (55.7) | 131 (44.3) | 1.0 |  | 1.0 |  |
|  | Yes | 452 (80.0) | 113 (20.0) | 3.17 (2.31, 4.4) | 0.000 | 1.92 (1.28, 2.87) | 0.001 |
| Sharing clothes | No | 302 (58.6) | 213 (41.4) | 1.0 |  | 1.0 |  |
|  | Yes | 315 (91.0) | 31 (9.0) | 9.78 (6.21, 15.38) | 0.000 | 5.13 (3.09, 8.50) | 0.000 |
| Sharing combs | No | 115 (47.7) | 126 (52.3) | 1.0 |  | 1.0 |  |
|  | Yes | 502 (81.0) | 118 (19.0) | 5.04 (3.57, 7.11) | 0.000 | 3.45 (2.23, 5.34) | 0.000 |
| **Household factors** | |  |  |  |  |  |  |
| Wealth status | Poor | 223 (77.7) | 64 (22.3) | 1.79 (1.22, 2.64) | 0.003 | 1.71 (1.04, 2.79) | 0.035 |
|  | Middle-class | 210 (70.7) | 87 (29.3) | 1.12 (0.77, 1.65) | 0.548 | 1.12 (0.69, 1.83) | 0.643 |
|  | Rich | 184 (66.4) | 93 (33.6) | 1.0 |  | 1.0 |  |
| **School factors** | |  |  |  |  |  |  |
| Access to health education on personal hygiene | Yes | 472 (70.0) | 202 (30.0) | 0.75 (0.46, 1.21) | 0.235 | 1.03 (0.54, 1.94) | 0.932 |
|  | No | 145 (77.5) | 42 (22.5) | 1.0 |  | 1.0 |  |
| **Variation and model fitness** | |  | | | **Final multivariate model** | | |
| Variance | School |  | | | 3.23E-31 | | |
|  | Class |  | | | 0.319 | | |
| Intra-cluster correlation coefficient | School |  | | | 8.95E-32 | | |
|  | Class |  | | | 8.85 | | |
| AIC |  |  | | | 722.9 | | |

CI: confidence interval; OR: odds ratio
