# Supplementary material for: Risk factors for scabies, tungiasis, and tinea infections among schoolchildren in southern Ethiopia: A cross-sectional Bayesian multilevel model
Source: PLoS Negl Trop Dis. 2021 Oct 6;15(10):e0009816. doi: 10.1371/journal.pntd.0009816 (PMC8494366; doi:10.1371/journal.pntd.0009816)
Supplement: S5 Table — (DOCX) [file pntd.0009816.s008.docx]

**S5 Table. Bivariate and multivariate, multilevel, mixed-effect, logistic regression analysis of scabies among schoolchildren in the Wonago district, southern Ethiopia, 2017**

| **Variables** | | **Scabies** | | | | | |
| --- | --- | --- | --- | --- | --- | --- | --- |
|  |  | **Yes (n (%)** | **No (n (%)** | **Crude odds ratio (COR) (95% CI)** | **P-value** | **Adjusted OR (95% CI)** | **P-value** |
| **Individual child factors** | |  |  |  |  |  |  |
| Sex | Boys | 31 (6.4) | 452 (93.6) | 1. 79 (0.93, 3.48) | 0.083 | 2.07 (1.02, 4.23) | 0.045 |
|  | Girls | 15 (4.0) | 363 (96.0) | 1.0 |  |  |  |
| Age in years (continuous) | Mean (SD) |  |  | 0.83 (0.66, 1.03) | 0.092 | 0.87 (0.68, 1.13) | 0.305 |
| Frequency of washing body with soap | Once per week | 24 (4.9) | 468 (95.1) | 1.0 |  | 1.0 |  |
|  | Every two weeks | 22 (6.0) | 347 (94.0) | 1.17 (0.62, 2.19) | 0.633 | 0.92 (0.46, 1.83) | 0.815 |
| Frequency of washing hair with soap | Once per week | 20 (4.3) | 446 (95.7) | 1.0 |  | 1.0 |  |
|  | Every two weeks | 26 (6.6) | 369 (93.4) | 1.61 (0.86, 3.03) | 0.136 | 1.18 (0.61, 2.29) | 0.621 |
| Frequency of washing legs and feet with soap | Once per day | 16 (3.9) | 391 (96.1) | 0.57 (0.29, 1.09) | 0.090 | 0.68 (0.34, 1.36) | 0.275 |
|  | Sometimes | 30 (6.6) | 424 (93.4) | 1.0 |  | 1.0 |  |
| Sharing beds | No | 7 (2.4) | 289 (97.6) | 1.0 |  |  |  |
|  | Yes | 39 (6.9) | 526 (93.1) | 3.39 (1.44, 8.02) | 0.005 | 2.97 (1.22, 7.21) | 0.016 |
| Sharing clothes | No | 22 (4.3) | 493 (95.7) | 1.0 |  | 1.0 |  |
|  | Yes | 24 (6.9) | 322 (93.1) | 1.52 (0.79, 2.91) | 0.211 | 1.03 (0.51, 2.09) | 0.926 |
| Sharing combs | No | 5 (2.1) | 236 (97.9) | 1.0 |  | 1.0 |  |
|  | Yes | 41 (6.6) | 579 (93.4) | 3.65 (1.37, 9.71) | 0.010 | 3.68 (1.31, 10.3) | 0.013 |
| **Household factors** | |  |  |  |  |  |  |
| Family size (continuous) | Mean (SD) |  |  | 1.14 (0.95, 1.36) | 0.163 | 1.16 (0.96, 1.40) | 0.127 |
| Wealth status | Poor | 16 (5.6) | 271 (94.4) | 1.37 (0.60, 3.10) | 0.452 | 1.08 (0.46, 2.57) | 0.856 |
|  | Middle-class | 18 (6.1) | 279 (93.9) | 1.44 (0.62, 3.35) | 0.391 | 1.28 (0.54, 3.04) | 0.581 |
|  | Rich | 12 (4.3) | 265 (95.7) | 1.0 |  | 1.0 |  |
| **School factors** | |  |  |  |  |  |  |
| Access to health education on personal hygiene | Yes | 26 (3.9) | 648 (96.1) | 0.48 (0.18, 1.30) | 0.151 | 0.49 (0.17, 1.38) | 0.177 |
|  | No | 20 (10.7) | 167 (89.3) | 1.0 |  |  |  |
| **Variation and model fitness** | |  | | | **Final multivariate model** | | |
| Variance | School |  | | | 0.369 | | |
|  | Class |  | | | 0.914 | | |
| Intra-cluster correlation coefficient | School |  | | | 8.1% | | |
|  | Class |  | | | 28% | | |
| AIC |  |  | | | 333.8 | | |

CI: confidence interval; OR: odds ratio
