# Supplementary material for: Risk factors for scabies, tungiasis, and tinea infections among schoolchildren in southern Ethiopia: A cross-sectional Bayesian multilevel model
Source: PLoS Negl Trop Dis. 2021 Oct 6;15(10):e0009816. doi: 10.1371/journal.pntd.0009816 (PMC8494366; doi:10.1371/journal.pntd.0009816)
Supplement: S6 Table — (DOCX) [file pntd.0009816.s009.docx]

**S6 Table. Bivariate and multivariate, multilevel, mixed-effect, logistic regression analysis of tungiasis among schoolchildren in the Wonago district, southern Ethiopia, 2017**

| **Variables** | | **Tungiasis** | | | | | |
| --- | --- | --- | --- | --- | --- | --- | --- |
|  |  | **Yes (n (%)** | **No (n (%)** | **Crude odds ratio (COR) (95% CI)** | **P-value** | **Adjusted OR (95% CI)** | **P-value** |
| **Individual child factors** | |  |  |  |  |  |  |
| Sex | Boys | 274 (56.7) | 209 (43.3) | 1.26 (0.95, 1.65) | 0.103 | 0.98 (0.72, 1.32) | 0.890 |
|  | Girls | 194 (51.3) | 184 (48.7) | 1.0 |  | 1.0 |  |
| Age in years (continuous) | Mean (SD) |  |  | 0.99 (0.91, 1.07) | 0.754 | 1.01 (0.92, 1.11) | 0.790 |
| Finger nails trimmed | Yes | 373 (52.9) | 332 (47.1) | 0.72 (0.50, 1.04) | 0.076 | 0.76 (0.51, 1.13) | 0.178 |
|  | No | 95 (60.9) | 61 (39.1) | 1.0 |  | 1.0 |  |
| Habit of walking barefoot | Always in barefoot | 11 (50.0) | 11 (50.0) | 0.92 (0.38, 2.20) | 0.847 | 0.54 (0.21, 1.42) | 0.211 |
|  | Sometimes in barefoot | 225 (56.3) | 175 (43.7) | 1.21 (0.90, 1.62) | 0.205 | 1.13 (0.82, 1.56) | 0.444 |
|  | Never in barefoot | 232 (52.8) | 207 (47.2) | 1.0 |  | 1.0 |  |
| Presence of footwear during exam | Yes | 459 (54.8) | 379 (45.2) | 2.06 (0.86, 4.93) | 0.105 | 2.07 (0.80, 5.37) | 0.134 |
|  | No | 9 (39.1) | 14 (60.9) | 1.0 |  | 1.0 |  |
| Frequency of washing body with soap | Once per week | 245 (49.8) | 247 (50.2) | 1.0 |  | 1.0 |  |
|  | Every two weeks | 223 (60.4) | 146 (39.6) | 1.56 (1.18, 2.07) | 0.002 | 1.41 (1.03, 1.93) | 0.031 |
| Frequency of washing legs and feet with soap | Once per day | 165 (40.5) | 242 (59.5) | 0.34 (0.26, 0.45) | 0.000 | 0.41 (0.30, 0.56) | 0.000 |
|  | Sometimes | 303 (66.7) | 151 (33.3) | 1.0 |  | 1.0 |  |
| Sharing beds | No | 122 (41.2) | 174 (58.8) | 1.0 |  | 1.0 |  |
|  | Yes | 346 (61.2) | 219 (38.8) | 2.26 (1.69, 3.03) | 0.000 | 1.83 (1.34, 2.51) | 0.000 |
| Sharing clothes | No | 230 (44.7) | 285 (55.3) | 1.0 |  | 1.0 |  |
|  | Yes | 238 (68.8) | 108 (31.2) | 3.13 (2.29, 4.29) | 0.000 | 2.39 (1.72, 3.32) | 0.000 |
| **Household factors** | |  |  |  |  |  |  |
| Family size | 1–4 | 45 (57.7) | 33 (42.3) | 1.0 |  | 1.0 |  |
|  | ≥5 | 423 (54.0) | 360 (46.0) | 0.84 (0.52, 1.36) | 0.490 | 0.80 (0.48, 1.35) | 0.412 |
| Wealth status | Poor | 174 (60.6) | 113 (39.4) | 1.61 (1.14, 2.26) | 0.006 | 1.51 (1.04, 2.20) | 0.031 |
|  | Middle-class | 158 (53.2) | 139 (46.8) | 1.14 (0.81, 1.61) | 0.446 | 1.08 (0.74, 1.58) | 0.674 |
|  | Rich | 136 (49.1) | 141 (50.9) | 1.0 |  | 1.0 |  |
| **School factors** | |  |  |  |  |  |  |
| Access to health education on personal hygiene | Yes | 364 (54.0) | 310 (46.0) | 0.97 (0.66, 1.41) | 0.855 | 1.03 (0.68, 1.58) | 0.877 |
|  | No | 104 (55.6) | 83 (44.4) | 1.0 |  | 1.0 |  |
| **Variation and model fitness** | |  | | | **Full multivariate model** | | |
| Variation | School |  | | | 5.77e-37 | | |
|  | Class |  | | | 0.096 | | |
| Intra-cluster correlation coefficient | School |  | | | 1.71e-37 | | |
|  | Class |  | | | 2.8% | | |
| AIC |  |  | | | 1087 | | |

CI: confidence interval; OR: odds ratio
