# Supplementary material for: Risk factors for scabies, tungiasis, and tinea infections among schoolchildren in southern Ethiopia: A cross-sectional Bayesian multilevel model
Source: PLoS Negl Trop Dis. 2021 Oct 6;15(10):e0009816. doi: 10.1371/journal.pntd.0009816 (PMC8494366; doi:10.1371/journal.pntd.0009816)
Supplement: S7 Table — (DOCX) [file pntd.0009816.s010.docx]

**S7 Table. Bivariate and multivariate multilevel, mixed-effect, logistic regression analysis of tinea infections among schoolchildren in the Wonago district, southern Ethiopia, 2017**

| **Variables** | | **Tinea infections** | | | | | |
| --- | --- | --- | --- | --- | --- | --- | --- |
|  |  | **Yes (n (%)** | **No (n (%)** | **Crude odds ratio (COR) (95% CI)** | **P-value** | **Adjusted OR (95% CI)** | **P-value** |
| **Individual child factors** | |  |  |  |  |  |  |
| Sex | Boys | 226 (46.8) | 257 (53.2) | 2.07 (1.55, 2.76) | 0.000 | 1.92 (1.40, 2.63) | 0.000 |
|  | Girls | 111 (29.4) | 267 (70.6) | 1.0 |  | 1.0 |  |
| Age in years (continuous ) | Mean (SD) |  |  | 0.99 (0.90, 1.08) | 0.793 | 1.02 (0.92, 1.13) | 0.697 |
| Unclean fingernails | Yes | 106 (50.7) | 103 (49.3) | 2.01 (1.43, 2.81) | 0.000 | 2.02 (1.40, 2.92) | 0.000 |
|  | No | 231 (35.4) | 421 (64.6) | 1.0 |  | 1.0 |  |
| Frequency of washing body with soap | Once per week | 183 (37.2) | 309 (62.8) | 1.0 |  | 1.0 |  |
|  | Every two weeks | 154 (41.7) | 215 (58.3) | 1.26 (0.95, 1.68) | 0.115 | 1.06 (0.76, 1.46) | 0.741 |
| Frequency of washing hair with soap | Once per week | 153 (32.8) | 313 (67.2) | 1.0 |  | 1.0 |  |
|  | Every two weeks | 184 (46.6) | 211 (53.4) | 1.81 (1.37, 2.41) | 0.000 | 1.53 (1.13, 2.08) | 0.006 |
| Frequency of washing legs and feet with soap | Once per day | 126 (31.0) | 281 (69.0) | 0.50 (0.37, 0.67) | 0.000 | 0.66 (0.48, 0.91) | 0.011 |
|  | Sometimes | 211 (46.5) | 243 (53.5) | 1.0 |  | 1.0 |  |
| Sharing beds | No | 84 (28.4) | 212 (71.6) | 1.0 |  | 1.0 |  |
|  | Yes | 253 (44.8) | 312 (55.2) | 2.09 (1.53, 2.85) | 0.000 | 1.48 (1.06, 2.08) | 0.021 |
| Sharing clothes | No | 158 (30.7) | 357 (69.3) | 1.0 |  | 1.0 |  |
|  | Yes | 179 (51.7) | 167 (48.3) | 2.61 (1.92, 3.53) | 0.000 | 1.63 (1.17, 2.28) | 0.004 |
| Sharing combs | No | 62 (25.7) | 179 (74.3) | 1.0 |  | 1.0 |  |
|  | Yes | 275 (44.4) | 345 (55.6) | 2.52 (1.78, 3.56) | 0.000 | 2.15(1.46, 3.17) | 0.000 |
| **Household factors** | |  |  |  |  |  |  |
| Family size | 1-4 | 27 (34.6) | 51 (65.4) | 1.0 |  | 1.0 |  |
|  | ≥5 | 310 (39.6) | 473 (60.4) | 1.25 (0.76, 2.06) | 0.376 | 1.24 (0.73, 2.12) | 0.428 |
| Wealth status | Poor | 119 (41.5) | 168 (58.5) | 1.34 (0.95, 1.91) | 0.100 | 1.19 (0.81, 1.75) | 0.379 |
|  | Middle-class | 120 (40.4) | 177 (59.6) | 1.22 (0.85, 1.74) | 0.286 | 1.23 (0.84, 1.81) | 0.285 |
|  | Rich | 98 (35.4) | 179 (64.6) | 1.0 |  | 1.0 |  |
| **School factors** | |  |  |  |  |  |  |
| Access to health education on personal hygiene | Yes | 253 (37.5) | 421 (62.5) | 0.80 (0.53, 1.20) | 0.283 | 0.91 (0.58, 1.45) | 0.706 |
|  | No | 84 (44.9) | 103 (55.1) | 1.0 |  | 1.0 |  |
| **Variation and model fitness** | |  | | | **Final multivariate model** | | |
| Variance | School |  | | | 9.01e-35 | | |
|  | Class |  | | | 0.171 | | |
| Intra-cluster correlation coefficient | School |  | | | 2.60e-35 | | |
|  | Class |  | | | 4.9% | | |
| AIC |  |  | | | 1055 | | |

CI: confidence interval; OR: odds ratio
