# Supplementary material for: Risk factors for scabies, tungiasis, and tinea infections among schoolchildren in southern Ethiopia: A cross-sectional Bayesian multilevel model
Source: PLoS Negl Trop Dis. 2021 Oct 6;15(10):e0009816. doi: 10.1371/journal.pntd.0009816 (PMC8494366; doi:10.1371/journal.pntd.0009816)
Supplement: S8 Table — (DOCX) [file pntd.0009816.s011.docx]

**S8 Table. Bayesian multivariate, multilevel, mixed-effect, logistic regression analysis of skin problem among schoolchildren in the Wonago district, southern Ethiopia, 2017**

| **Variables** | | **Any skin problems** | | | | |
| --- | --- | --- | --- | --- | --- | --- |
|  |  | **Posterior mean** | **SD** | **MCSE** | **Median** | **Adjusted 95% Bayesian credible intervals (BCI) OR (95% BCI)** |
| **Individual child factors** | |  |  |  |  |  |
| Sex | Boys | 1.55 | 0.325 | 0.003 | 1.52 | 1.55 (1.01, 2.28)* |
|  | Girls | - | - | - | - | 1.0 |
| Age in years | Mean (SD) | 1.02 | 0.068 | 0.004 | 1.01 | 1.02 (0.89, 1.15) |
| Unclean fingernails | Yes | 1.85 | 0.48 | 0.004 | 1.79 | 1.85 (1.08, 2.97)* |
|  | No | - | - | - | - | 1.0 |
| Presence of footwear during exam | Yes | 2.68 | 1.63 | 0.057 | 2.29 | 2.68 (0.74, 6.91) |
|  | No | - | - | - | - | 1.0 |
| Frequency of washing body with soap | Once per week | - | - | - | - | 1.0 |
|  | Every two weeks | 1.90 | 0.42 | 0.005 | 1.85 | 1.90 (1.21, 2.85)* |
| Frequency of washing hair with soap | Once per week | - | - | - | - | 1.0 |
|  | Every two weeks | 3.07 | 0.66 | 0.007 | 3.00 | 3.07 (1.98, 4.57* |
| Frequency of washing legs and feet with soap | Once per day | 0.23 | 0.048 | 0.0007 | 0.22 | 0.23 (0.15, 0.33)* |
|  | Sometimes | - | - | - | - | 1.0 |
| Sharing beds | No | - | - | - | - | 1.0 |
|  | Yes | 1.97 | 0.42 | 0.005 | 1.93 | 1.97 (1.27, 2.89)* |
| Sharing clothes | No | - | - | - | - | 1.0 |
|  | Yes | 5.65 | 1.51 | 0.021 | 5.44 | 5.65 (3.31, 9.21)* |
| Sharing combs | No | - | - | - | - | 1.0 |
|  | Yes | 3.65 | 0.84 | 0.011 | 3.57 | 3.65 (2.28, 5.53)* |
| **Household factors** | |  |  |  |  |  |
| Wealth status | Poor | 1.76 | 0.46 | 0.005 | 1.70 | 1.76 (1.03, 2.83)* |
|  | Middle-class | 1.13 | 0.29 | 0.004 | 1.09 | 1.13 (0.66, 1.79) |
|  | Rich | - | - | - | - | 1.0 |
| **School factors** | |  |  |  |  |  |
| Access to health education on personal hygiene | Yes | 1.26 | 0.49 | 0.009 | 1.17 | 1.26 (0.58, 2.44) |
|  | No | - | - | - | - | 1.0 |
| **Variation And model fitness** | |  | | | **Final multivariate model** | |
| Variation | School |  | | | 0.206 | |
|  | Class |  | | | 0.419 | |
| Intra-cluster correlation coefficient | School |  | | | 5.3% | |
|  | Class |  | | | 16.0% | |
| DIC |  |  | | | 712.8 | |

BCI: Bayesian credible interval; OR: odds ratio; SD: standard deviations; MCSE: Monte Carlo standard errors; *significant
