# Supplementary material for: Risk factors for scabies, tungiasis, and tinea infections among schoolchildren in southern Ethiopia: A cross-sectional Bayesian multilevel model
Source: PLoS Negl Trop Dis. 2021 Oct 6;15(10):e0009816. doi: 10.1371/journal.pntd.0009816 (PMC8494366; doi:10.1371/journal.pntd.0009816)
Supplement: S9 Table — (DOCX) [file pntd.0009816.s012.docx]

**S9 Table. Bayesian multivariate, multilevel, mixed-effect, logistic regression analysis of scabies among schoolchildren in the Wonago district, southern Ethiopia, 2017**

| **Variables** | | **Scabies** | | | | |
| --- | --- | --- | --- | --- | --- | --- |
|  |  | **Posterior mean** | **SD** | **MCSE** | **Median** | **Adjusted 95% Bayesian credible intervals (BCI) OR (95% BCI)** |
| **Individual child factors** | |  |  |  |  |  |
| Sex | Boys | 2.62 | 1.05 | 0.014 | 2.42 | 2.62 (1.19, 5.19)* |
|  | Girls | - | - | - | - | 1.0 |
| Age in years (continous) | Mean (SD) | 0.82 | 0.09 | 0.005 | 0.81 | 0.82 (0.66, 1.04) |
| Frequency of washing body with soap | Every week | - | - | - | - | 1.0 |
|  | Every two weeks | 1.15 | 0.43 | 0.006 | 1.08 | 1.15 (0.52, 2.18) |
| Frequency of washing hair with soap | Every week | - | - | - | - | 1.0 |
|  | Every two weeks | 1.41 | 0.52 | 0.006 | 1.33 | 1.41 (0.68, 2.64) |
| Frequency of washing legs and feet with soap | Every day | 0.88 | 0.33 | 0.005 | 0.83 | 0.88 (0.41, 1.65) |
|  | Sometimes | - | - | - | - | 1.0 |
| Sharing beds | No | - | - | - | - | 1.0 |
|  | Yes | 4.30 | 2.36 | 0.05 | 3.74 | 4.30 (1.55, 10.4)* |
| Sharing clothes | No | - | - | - | - | 1.0 |
|  | Yes | 1.25 | 0.48 | 0.006 | 1.17 | 1.25 (0.56, 2.39) |
| Sharing combs | No | - | - | - | - | 1.0 |
|  | Yes | 5.77 | 3.93 | 0.08 | 4.75 | 5.77 (1.72, 16.1)* |
| **Household factors** | |  |  |  |  |  |
| Family size (continuous) | Mean (SD) | 1.15 | 0.11 | 0.003 | 1.15 | 1.15 (0.94, 1.39) |
| Wealth status | Poor | 1.68 | 0.85 | 0.011 | 1.50 | 1.68 (0.61, 3.79) |
|  | Middle-class | 1.92 | 0.96 | 0.014 | 1.72 | 1.96 (0.70, 4.32) |
|  | Rich | - | - | - | - | 1.0 |
| **School factors** | |  |  |  |  |  |
| Access to health education on personal hygiene | Yes | 0.77 | 0.46 | 0.008 | 0.66 | 0.77 (0.24, 1.95) |
|  | No | - | - | - | - | 1.0 |
| **Variation and model fitness** | |  | | | **Final multivariate model** | |
| Variation | School |  | | | 2.29 | |
|  | Class |  | | | 1.16 | |
| Intra-cluster correlation coefficient | School |  | | | 31.2% | |
|  | Class |  | | | 49.3% | |
| DIC |  |  | | | 321 | |

BCI: Bayesian credible interval; OR: odds ratio; SD: standard deviations; MCSE: Monte Carlo standard errors; *significant
