# Supplementary material for: Risk factors for scabies, tungiasis, and tinea infections among schoolchildren in southern Ethiopia: A cross-sectional Bayesian multilevel model
Source: PLoS Negl Trop Dis. 2021 Oct 6;15(10):e0009816. doi: 10.1371/journal.pntd.0009816 (PMC8494366; doi:10.1371/journal.pntd.0009816)
Supplement: S10 Table — (DOCX) [file pntd.0009816.s013.docx]

**S10 Table.** **Bayesian multivariate, multilevel, mixed-effect, logistic regression analysis of tungiasis among schoolchildren in the Wonago district, southern Ethiopia, 2017**

| **Variables** | | **Tungiasis** | | | | |
| --- | --- | --- | --- | --- | --- | --- |
| **Individual child factors** | | **Posterior mean** | **SD** | **MCSE** | **Median** | **Adjusted 95% Bayesian credible intervals (BCI) OR (95% BCI)** |
| Sex | Boys | 1.08 | 0.17 | 0.002 | 1.07 | 1.08 (0.78, 1.46) |
|  | Girls | - | - | - | - | 1.0 |
| Age in years | Mean (SD) | 1.01 | 0.05 | 0.002 | 1.01 | 1.01 (0.91, 1.11) |
| Finger nails trimmed | Yes | 0.93 | 0.20 | 0.003 | 0.91 | 0.93 (0.60, 1.38) |
|  | No | - | - | - | - | 1.0 |
| Habit of walking barefoot | Always in barefoot | 2.41 | 1.53 | 0.027 | 2.03 | 2.41 (0.71, 6.39) |
|  | Sometimes in barefoot | 1.40 | 0.24 | 0.003 | 1.38 | 1.40 (0.99, 1.93) |
|  | Never in barefoot | - | - | - | - | 1.0 |
| Presence of footwear during exam | Yes | 8.04 | 4.98 | 0.29 | 6.76 | 8.04 (2.46, 21.4)* |
|  | No | - | - | - | - | 1.0 |
| Frequency of washing body with soap | Every week | - | - | - | - | 1.0 |
|  | Every two weeks | 1.59 | 0.26 | 0.003 | 1.57 | 1.59 (1.13, 2.17)* |
| Frequency of washing legs and feet with soap | Every day | 0.48 | 0.08 | 0.0009 | 0.48 | 0.48 (0.35, 0.65)* |
|  | Sometimes | - | - | - | - | 1.0 |
| Sharing beds | No | - | - | - | - | 1.0 |
|  | Yes | 2.00 | 0.33 | 0.004 | 1.97 | 2.00 (1.43, 2.71)* |
| Sharing clothes | No | - | - | - | - | 1.0 |
|  | Yes | 2.85 | 0.50 | 0.006 | 2.80 | 2.85 (2.01, 3.97)* |
| **Household factors** | |  |  |  |  |  |
| Family size | 1-4 | - | - | - | - | 1.0 |
|  | ≥5 | 1.02 | 0.28 | 0.005 | 0.98 | 1.02 (0.58, 1.66) |
| Wealth status | Poor | 1.93 | 0.39 | 0.005 | 1.89 | 1.93 (1.29, 2.79)* |
|  | Middle-class | 1.36 | 0.27 | 0.004 | 1.34 | 1.36 (0.89, 1.98) |
|  | Rich | - | - | - | - | 1.0 |
| **School factors** | |  |  |  |  |  |
| Access to health education on personal hygiene | Yes | 1.52 | 0.43 | 0.008 | 1.45 | 1.52 (0.87, 2.54) |
|  | No | - | - | - | - | 1.0 |
| **Variation and model fitness** | |  | | | **Full multivariate model** | |
| Variation | School |  | | | 0.106 | |
|  | Class |  | | | 0.198 | |
| Intra-cluster correlation coefficient | School |  | | | 3% | |
|  | Class |  | | | 8.5% | |
| DIC |  |  | | | 1098 | |

BCI: Bayesian credible interval; OR: odds ratio; SD: standard deviations; MCSE: Monte Carlo standard errors; *significant
