# Supplementary material for: Risk factors for scabies, tungiasis, and tinea infections among schoolchildren in southern Ethiopia: A cross-sectional Bayesian multilevel model
Source: PLoS Negl Trop Dis. 2021 Oct 6;15(10):e0009816. doi: 10.1371/journal.pntd.0009816 (PMC8494366; doi:10.1371/journal.pntd.0009816)
Supplement: S11 Table — (DOCX) [file pntd.0009816.s014.docx]

**S11 Table. Bayesian multivariate, multilevel, mixed-effect, logistic regression analysis of tinea infections among schoolchildren in the Wonago district, southern Ethiopia, 2017**

| **Variables** | | **Tinea infections** | | | | |
| --- | --- | --- | --- | --- | --- | --- |
| **Individual child factors** | | **Posterior mean** | **SD** | **MCSE** | **Median** | **Adjusted 95% Bayesian credible intervals (BCI) OR (95% BCI)** |
| Sex | Boys | 2.42 | 0.42 | 0.004 | 2.38 | 2.42 (1.72, 3.34)* |
|  | Girls | - | - | - | - | 1.0 |
| Age in years (continous) | Mean (SD) | 1.05 | 0.058 | 0.003 | 1.04 | 1.04 (0.94, 1.16) |
| Unclean fingernails | Yes | 2.96 | 0.60 | 0.006 | 2.89 | 2.96 (1.96, 4.31)* |
|  | No | - | - | - | - | - |
| Frequency of washing body with soap | Every week | - | - | - | - | 1.0 |
|  | Every two weeks | 1.36 | 0.24 | 0.003 | 1.34 | 1.36 (0.95, 1.89) |
| Frequency of washing hair with soap | Once per week | - | - | - | - | 1.0 |
|  | Every two weeks | 1.94 | 0.32 | 0.005 | 1.92 | 1.94 (1.39, 2.66)* |
| Frequency of washing legs and feet with soap | Every day | 0.89 | 0.15 | 0.002 | 0.88 | 0.89 (0.63, 1.23) |
|  | Sometimes | - | - | - | - | 1.0 |
| Sharing beds | No | - | - | - | - | 1.0 |
|  | Yes | 1.78 | 0.33 | 0.004 | 1.74 | 1.78 (1.22, 2.51)* |
| Sharing clothes | No | - | - | - | - | 1.0 |
|  | Yes | 1.89 | 0.35 | 0.004 | 1.86 | 1.89 (1.30, 2.65)* |
| Sharing combs | No | 2.93 | 0.63 | 0.011 | 2.86 | 2.93 (1.90, 4.37)* |
|  | Yes | - | - | - | - | - |
| **Household factors** | |  |  |  |  |  |
| Family size | 1-4 | - | - | - | - | 1.0 |
|  | ≥5 | 2.25 | 0.72 | 0.015 | 2.13 | 2.25 (1.20, 3.92) |
| Wealth status | Poor | 1.99 | 0.42 | 0.006 | 1.94 | 1.99 (1.29, 2.94)* |
|  | Middle-class | 2.01 | 0.43 | 0.006 | 1.97 | 2.01 (1.30, 2.98)* |
|  | Rich | - | - | - | - | 1.0 |
| **School factors** | |  |  |  |  |  |
| Access to health education on personal hygiene | Yes | 1.11 | 0.32 | 0.006 | 1.06 | 1.11 (0.62, 1.88) |
|  | No | - | - | - | - | 1.0 |
| **Variation and model fitness** | |  | | | **Final multivariate model** | |
| Variance | School |  | | | 0.217 | |
|  | Class |  | | | 0.264 | |
| Intra-cluster correlation coefficient | School |  | | | 5.7% | |
|  | Class |  | | | 12.7% | |
| DIC |  |  | | | 1071 | |

BCI: bayesian credible interval; OR: odds ratio; SD: standard deviations; MCSE: Monte Carlo standard errors; *significant
