# Supplementary material for: Risk factors for scabies, tungiasis, and tinea infections among schoolchildren in southern Ethiopia: A cross-sectional Bayesian multilevel model
Source: PLoS Negl Trop Dis. 2021 Oct 6;15(10):e0009816. doi: 10.1371/journal.pntd.0009816 (PMC8494366; doi:10.1371/journal.pntd.0009816)
Supplement: Attachment — Submitted filename: Response to Reviewers.docx [file pntd.0009816.s015.docx]

**Tracking number: PNTD-D-21-01055**

**Title: "Risk factors for scabies, tungiasis, and tinea infections among schoolchildren in southern Ethiopia: A cross-sectional Bayesian multilevel model"**

**Responses to reviewers’ comments and suggestions:**

| **Reviewer's Responses to Questions** | **Author’s reply** |
| --- | --- |
|  | We thank the reviewer #1 and #2 for the positive and very helpful comments and suggestions. |
| **Methods** |  |
| **Reviewer #1:** (No Response) |  |
| **Reviewer #2:** |  |
| -Are the objectives of the study clearly articulated with a clear testable hypothesis stated? YES  -Is the study design appropriate to address the stated objectives? YES  -Is the population clearly described and appropriate for the hypothesis being tested? YES  -Is the sample size sufficient to ensure adequate power to address the hypothesis being tested? YES  -Were correct statistical analysis used to support conclusions? YES  -Are there concerns about ethical or regulatory requirements being met? YES | We thank the reviewer #2 for the positive comments. |
| **Results** |  |
| **Reviewer #1:** (No Response) |  |
| **Reviewer #2:** |  |
| -Does the analysis presented match the analysis plan? YES  -Are the results clearly and completely presented? YES  -Are the figures (Tables, Images) of sufficient quality for clarity? YES | We thank the reviewer #2 for the positive comments. |
| **Conclusions** |  |
| **Reviewer #1:** (No Response) |  |
| **Reviewer #2:** |  |
| -Are the conclusions supported by the data presented? YES  -Are the limitations of analysis clearly described? YES  -Do the authors discuss how these data can be helpful to advance our understanding of the topic under study? YES  -Is public health relevance addressed? YES | We thank the reviewer #2 for the positive comments. |
| **Editorial and Data Presentation Modifications?** |  |
| Reviewer #1: (No Response) |  |
| Reviewer #2: (No Response) |  |
| **Summary and General Comments** |  |
| **Reviewer #1:** |  |
| I read with interest the manuscript entitled “Risk factors for scabies, tungiasis, and tinea infections among schoolchildren in southern Ethiopia: A cross-sectional Bayesian multilevel model”.  The manuscript is well written, the methods are sound, the results clearly presented, and the conclusion relevant. The authors identified several risk factors for skin conditions, including low socioeconomic status; unclean fingernails; not washing with soap at least every week; and sharing beds, clothes, and combs. They conclude that improving the personal hygiene of schoolchildren through education is important.  The main strength of this study are the random inclusion procedure and the relatively high number of children studied, which make its findings generalizable to school-aged children in similar socio-economic and cultural settings. The use of both frequentist and Bayesian models and the fact that both yield similar results is also convincing.  As discussed by the authors this study limitation was that the diagnosis of each studied condition was only clinical and no laboratory confirmation test was performed. Yet the clinical signs of the studied skin diseases are relatively characteristic and thus possible selection and/or misclassification biases are likely to be marginal.  I have a minor remark: To improve the manuscript readability, I would suggest detailing in the text of the results section only one analysis results (for instance, only the Bayesian and not the frequentist analyses results). Because both analyses yielded similar results, which are detailed in the corresponding Tables. | We thank the reviewer #1 for your positive and very helpful suggestions.    In the result section we have removed the text of the frequentist result as suggested. We did the detailing of text only for Bayesian approach accordingly. |
| **Reviewer #2:** |  |
| General comments |  |
| This is a carefully designed study on the prevalence and of the risk factors of tinea, scabies and tungiasis among school children in Southern Ethiopia.  The study is remarkable because multitude of risk factors were assessed with both, a multilevel, mixed-effect, logistic regression and a Bayesian multilevel, mixed-effect, logistic regression. These three skin diseases are associated with important disease burden in sub-Saharan Africa and are neglected by health care providers. | We thank the reviewer #2 for your very helpful suggestions and comments. |
| **Specific comments** |  |
| Line 35-36: Rephrase sentence as follows: “Washing….. daily was identified as a protective factor for each of the three skin diseases.” | We have rephrased the suggested sentence in Page 2; lines 35-36: Washing legs and feet with soap daily was identified as a protective factor for each of the three skin diseases |
| Line 59: Tungiasis is not an infestation, but an infection. Change “infestation” to “infection” in the whole text. Tungiasis (in some countries in Africa called “jiggers”) is the disease, where as T. penetrans, the sand flea (in Africa called the “jigger flea”) is the pathogen. | Thank you for the comment, we have revised throughout the manuscript.  infestation changed to “infection” |
| Line 60: Change to “…. is estimated to be up to 50% in some endemic areas” | Thank you for the comment. We have revised in page 4; line 61: The prevalence of tungiasis (Jigger flea) infections is estimated to be up to 50% in some endemic areas |
| Line 66: Scabies is not a dermatosis but an ectoparasitic skin disease. Lesions may occur at any part of the body, the topographic distribution depending on age. The pathogen is Sarcoptes scabiei var. hominis. | Thank you for the comments, we have revised in page:4; lines: 67-69:  Scabies, an intensely itchy ectoparasitic skin disease, results from infestations of the mite *Sarcoptes scabiei var. hominis* [5,6]. Scabies lesions can occur in several body sites, but the topographic distribution of scabies lesions may vary with age [6-9]. |
| Line 70-72: Differentiate between symptoms and signs of acute and chronic manifestations of tungiasis and the socio-economic impact the disease may have. | We agreed that all suggested information revised in detail in the introduction accordingly.  Page: 4: lines; 72-76: It causes erythema, edema tenderness, itching, and pain in acute phase; and shining skin, desquamation, nail and toes deformity or loss of nails may occur in the chronic phase [13]. It have also socio-economic impact that includes difficulty walking or gripping, sleep disturbances, stigma, and all of which affects quality of life, school absenteeism, and dropout rates [13-16]. |
| Line 73: Change into “Bacterial superinfection is almost constant. Death may ensue when bacterial superinfection causes tetanus, gangrene, or septicaemia.” | Thank you for the suggestion, we have changed in page: 4; lines; 76-78: Bacterial superinfection is almost constant. Death may ensue when bacterial superinfection causes tetanus, gangrene, or septicaemia [13,14,17]. |
| Line 74: The publication refers to the Wensho district not to Ethiopia in general. | Thank you for pointing it out. We have corrected in page: 4; lines: 78 -80: Ethiopia has a favorable environment for jigger fleas [18], and the prevalence of tungiasis ranges between 35% to 60% among school-aged children in southern Ethiopia [16,19]. |
| Line 81-88: Section could be shifted to the Discussion | Thank you for suggestion. However, the sentences in page: 5; lines 87-94 were included to explain the general services included in the school health program. Thus, this was just to indicate as assessment of skin problems are among one of school health services although major gap exist in the implementation process. Therefore, this could be better if we include in the introduction rather than shifting to the discussion section.  Lines: 87-94: Schoolchildren in Ethiopia are vulnerable to various health problems [23]. High rates of nutritional disorders, intestinal parasitic infections, and skin problems have been reported [24]. Although some school health programs include programs to address scabies, tungiasis, and fungal infections, including mandatory periodic screenings, most of these programs are non-functional [23]. In 2012, Ethiopia established the National School Health and Nutrition Strategy aiming to improve schoolchildren’s access to health interventions [25], such as nutrition [26], deworming [27], and water, sanitation, and hygiene [24]. These efforts are ongoing, but major gaps persist in the implementation of these school health services [23]. |
| Line 90: The cited publications refer to only one skin disease but not to a group of diseases. | Thank you for pointing it out. Revised in page:5; line:95: Most studies on skin problems among primary schoolchildren in Ethiopia did not assess them collectively [19,22,28,29] or focus on scabies [11,30-33]. |
| Line 95-103: Section belongs to the Discussion | We agreed that all suggested information moved to the Discussion in page: 22; lines: 379-387:  Unlike the frequentist model, a Bayesian model uses fixed data, and the estimated parameters are viewed as random. It also includes prior information and does not rely on sampling, instead using probability to indicate uncertainty in the model [34]. Interpreting credible intervals is easier in the Bayesian model than in the frequentist model because the range of values in the Bayesian approach has a direct probabilistic interpretation of the true estimate [34,35]. In our model, we clustered individual data within the same classroom and classrooms within the same school. To account for clustering effects, we applied a multilevel analysis using both the frequentist and Bayesian approaches. |
| Line 118-128: It remains unclear at which period of the year the study was done. The prevalence of tungiasis and scabies vary according to the season of the year (Jackson et al. 2007, Trop Med Int Health, doi: 10.1111/j.1365-3156.2006.01809.x). This may have an impact on the risk-factors assessed in this study and needs to be discussed. | Thank you for this comment. We have discussed in page: 24; lines: 430-438: Scabies varied with seasons [57,58,60]. Our study period was in dry season, this could also affect scabies prevalence because scabies is more common in cool and humid weather condition [58,60]. In contrast, other evidence indicates that an increase in temperature, is associated with increased scabies prevalence [57]. It has previously been reported that the highest cases of scabies in community based study in drought affected area in Ethiopia [30]. Though we did not assess the seasonal variations in this study due to the nature of our study design, it might be linked with overcrowding and close contact in the cooler seasons. Other studies, however, did not find significant variations with seasons [7,61,62].  In page 26; lines: 442-444: It also could be linked with seasonal variations because our study period was in dry season, where tungiasis is more common [2,64,65]. This may be explained with indirect link with access to water in the dry season for personal hygiene protection. |
| Line 170-175: The diagnosis of tungiasis has to take into account the characteristic development of an embedded female sand flea in the epidermis and the reaction of the host, and should be done according to the Fortaleza classification (Eisele et al. 2003, Parasitol Res, doi: 10.1007/s00436-002-0817-y). The clinical findings used as diagnostic criteria by the authors are a mixture of different parasitic stages, secondary complications (“surrounding tissue necrosis”) and manipulation of lesions by the patient or a caregiver (“crater-like score”). “Circular wounds in the keratin layer“ do not exist. The authors probably mean a circular impression in the epidermis corresponding to stage IV of the Fortaleza classification. Hence, the diagnosis of tungiasis was biased and will have resulted in an unknown number of false-positive and false-negative diagnoses and, by consequence, to an over-or-under-estimate of prevalence, respectively. | We acknowledge your concern. We used Fortaleza classification to diagnose tungiasis infection. We have stated in detail in page:8; lines:168-175  Tungiasis was diagnosed with Fortaleza classification via clinical findings of penetrating sand fleas: a small, dark, itchy spot in the epidermis with visible posterior parts of the parasite, with or without local pain; a brownish or black dot surrounded by erythema; a circular yellow-white patch with a central black dot representing the posterior segments of the parasite; a circular brownish-black crust with or without surrounding necrosis of the epidermis. We also recorded a manipulated lesions: a characteristic crater-like sore or lesion and suppurating lesions caused by the use of non-sterile perforating instruments (i.e. needles) [46]. |
| Line 293-296: Section belongs to Methods. | Thank you for this comment. We have moved to the method section in page: 12; lines: 251-255 |
| Line 407-408: The authors correctly state that the prevalence/incidence of scabies and tungiasis are influenced by the season of the year. For tungiasis the difference in prevalence between the end of the rainy season and the end of the dry season maybe up to 80 percent. Although the authors acknowledge this in line 423 they do not discuss the impact this will have on risk-factors which themselves are influenced by the season. For instance, in many endemic areas water supply is restricted in the dry season, and children may not be able to wash their feet even if soap is available. | Thank you for positive comment. We have indicated in the discussion section in page: 24; lines: 442-444: It also could be linked with seasonal variations because our study period was in dry season, where tungiasis is more common [2,64,65]. This may be explained with indirect link with access to water in the dry season for personal hygiene protection. |
| Line 423: Prevalence is not a rate but a proportion | Corrected in page: 25; line:426: Our finding of a 5% prevalence for scabies aligns with that of similar studies in southern Ethiopia [16] |
| Line 428: Prevalences between settings can only be compared if the studies were performed at the same season of the year. | Thank you for the suggestion. We compared with studies in comparable seasons. In page: 24; lines: 438-440: The prevalence of tungiasis was 54%, which aligns with other findings from rural southern Ethiopia [19] and Kenya [63] but exceeds the 35% found in other areas in southern Ethiopia [16]. |
| Line 443-445: Change to “Our results similarly show that washing the feet daily with soap was a protective factor of both, tungiasis and tinea. | Thank you. We have changed the suggested sentences in page: 25; lines: 456-458: Our results similarly show that washing the feet daily with soap was a protective factor of both, tungiasis and tinea. |
| Line 488: Change to “We found significant associations between many individual hygiene-related factors and tinea, scabies and tungiasis.” | Thank you. We have changed the suggested sentences in page: 27; lines: 499 and 500: We found significant associations between many individual hygiene-related factors and scabies, tungiasis and tinea. |
