# Supplementary material for: Risk factors for scabies, tungiasis, and tinea infections among schoolchildren in southern Ethiopia: A cross-sectional Bayesian multilevel model
Source: PLoS Negl Trop Dis. 2021 Oct 6;15(10):e0009816. doi: 10.1371/journal.pntd.0009816 (PMC8494366; doi:10.1371/journal.pntd.0009816)
Supplement: Peer review history

# Peer review: Decision Letter 0

**DOI:** 10.1371/journal.pntd.0009816.r001

**Authors:** Bruce Y. Lee, Uwem Friday Ekpo

16 Aug 2021

Dear Mrs Amare,

Thank you very much for submitting your manuscript "Risk factors for scabies, tungiasis, and tinea infections among schoolchildren in southern Ethiopia: A cross-sectional Bayesian multilevel model" for consideration at PLOS Neglected Tropical Diseases. As with all papers reviewed by the journal, your manuscript was reviewed by members of the editorial board and by several independent reviewers. The reviewers appreciated the attention to an important topic. Based on the reviews, we are likely to accept this manuscript for publication, providing that you modify the manuscript according to the review recommendations. 

Please prepare and submit your revised manuscript within 30 days. If you anticipate any delay, please let us know the expected resubmission date by replying to this email. 

When you are ready to resubmit, please upload the following:

[1] A letter containing a detailed list of your responses to all review comments, and a description of the changes you have made in the manuscript. 

Please note while forming your response, if your article is accepted, you may have the opportunity to make the peer review history publicly available. The record will include editor decision letters (with reviews) and your responses to reviewer comments. If eligible, we will contact you to opt in or out

[2] Two versions of the revised manuscript: one with either highlights or tracked changes denoting where the text has been changed; the other a clean version (uploaded as the manuscript file).

Important additional instructions are given below your reviewer comments. 

Thank you again for your submission to our journal. We hope that our editorial process has been constructive so far, and we welcome your feedback at any time. Please don't hesitate to contact us if you have any questions or comments.

Sincerely,

Uwem Friday Ekpo, PhD

Associate Editor

PLOS Neglected Tropical Diseases

Bruce Lee

Deputy Editor

PLOS Neglected Tropical Diseases

***********************

Reviewer's Responses to Questions

**Key Review Criteria Required for Acceptance?**

As you describe the new analyses required for acceptance, please consider the following:

**Methods**

-Are the objectives of the study clearly articulated with a clear testable hypothesis stated?

-Is the study design appropriate to address the stated objectives?

-Is the population clearly described and appropriate for the hypothesis being tested?

-Is the sample size sufficient to ensure adequate power to address the hypothesis being tested?

-Were correct statistical analysis used to support conclusions?

-Are there concerns about ethical or regulatory requirements being met?

Reviewer #1: (No Response)

Reviewer #2: -Are the objectives of the study clearly articulated with a clear testable hypothesis stated? YES

-Is the study design appropriate to address the stated objectives? YES

-Is the population clearly described and appropriate for the hypothesis being tested? YES

-Is the sample size sufficient to ensure adequate power to address the hypothesis being tested? YES

-Were correct statistical analysis used to support conclusions? YES

-Are there concerns about ethical or regulatory requirements being met? YES

--------------------

**Results**

-Does the analysis presented match the analysis plan?

-Are the results clearly and completely presented?

-Are the figures (Tables, Images) of sufficient quality for clarity?

Reviewer #1: (No Response)

Reviewer #2: -Does the analysis presented match the analysis plan? YES

-Are the results clearly and completely presented? YES

-Are the figures (Tables, Images) of sufficient quality for clarity? YES

--------------------

**Conclusions**

-Are the conclusions supported by the data presented?

-Are the limitations of analysis clearly described?

-Do the authors discuss how these data can be helpful to advance our understanding of the topic under study?

-Is public health relevance addressed?

Reviewer #1: (No Response)

Reviewer #2: -Are the conclusions supported by the data presented? YES

-Are the limitations of analysis clearly described? YES

-Do the authors discuss how these data can be helpful to advance our understanding of the topic under study? YES

-Is public health relevance addressed? YES

--------------------

**Editorial and Data Presentation Modifications?**

Use this section for editorial suggestions as well as relatively minor modifications of existing data that would enhance clarity. If the only modifications needed are minor and/or editorial, you may wish to recommend “Minor Revision” or “Accept”. 

Reviewer #1: (No Response)

Reviewer #2: (No Response)

--------------------

**Summary and General Comments**

Use this section to provide overall comments, discuss strengths/weaknesses of the study, novelty, significance, general execution and scholarship. You may also include additional comments for the author, including concerns about dual publication, research ethics, or publication ethics. If requesting major revision, please articulate the new experiments that are needed.

Reviewer #1: I read with interest the manuscript entitled “Risk factors for scabies, tungiasis, and tinea infections among schoolchildren in southern Ethiopia: A cross-sectional Bayesian multilevel model”.

The manuscript is well written, the methods are sound, the results clearly presented, and the conclusion relevant. The authors identified several risk factors for skin conditions, including low socioeconomic status; unclean fingernails; not washing with soap at least every week; and sharing beds, clothes, and combs. They conclude that improving the personal hygiene of schoolchildren through education is important.

The main strength of this study are the random inclusion procedure and the relatively high number of children studied, which make its findings generalizable to school-aged children in similar socio-economic and cultural settings. The use of both frequentist and Bayesian models and the fact that both yield similar results is also convincing. 

As discussed by the authors this study limitation was that the diagnosis of each studied condition was only clinical and no laboratory confirmation test was performed. Yet the clinical signs of the studied skin diseases are relatively characteristic and thus possible selection and/or misclassification biases are likely to be marginal. 

I have a minor remark: To improve the manuscript readability, I would suggest detailing in the text of the results section only one analysis results (for instance, only the Bayesian and not the frequentist analyses results). Because both analyses yielded similar results, which are detailed in the corresponding Tables.

Reviewer #2: General comments

This is a carefully designed study on the prevalence and of the risk factors of tinea, scabies and tungiasis among school children in Southern Ethiopia. 

The study is remarkable because multitude of risk factors were assessed with both, a multilevel, mixed-effect, logistic regression and a Bayesian multilevel, mixed-effect, logistic regression. These three skin diseases are associated with important disease burden in sub-Saharan Africa and are neglected by health care providers.

Specific comments

Line 35-36: Rephrase sentence as follows: “Washing….. daily was identified as a protective factor for each of the three skin diseases.”

Line 59: Tungiasis is not an infestation, but an infection. Change “infestation” to “infection” in the whole text. Tungiasis (in some countries in Africa called “jiggers”) is the disease, where as T. penetrans, the sand flea (in Africa called the “jigger flea”) is the pathogen.

Line 60: Change to “…. is estimated to be up to 50% in some endemic areas”

Line 66: Scabies is not a dermatosis but an ectoparasitic skin disease. Lesions may occur at any part of the body, the topographic distribution depending on age. The pathogen is Sarcoptes scabiei var. hominis.

Line 70-72: Differentiate between symptoms and signs of acute and chronic manifestations of tungiasis and the socio-economic impact the disease may have.

Line 73: Change into “Bacterial superinfection is almost constant. Death may ensue when bacterial superinfection causes tetanus, gangrene, or septicaemia.”

Line 74: The publication refers to the Wensho district not to Ethiopia in general.

Line 81-88: Section could be shifted to the Discussion

Line 90: The cited publications refer to only one skin disease but not to a group of diseases.

Line 95-103: Section belongs to the Discussion

Line 118-128: It remains unclear at which period of the year the study was done. The prevalence of tungiasis and scabies vary according to the season of the year (Jackson et al. 2007, Trop Med Int Health, doi: 10.1111/j.1365-3156.2006.01809.x). This may have an impact on the risk-factors assessed in this study and needs to be discussed.

Line 170-175: The diagnosis of tungiasis has to take into account the characteristic development of an embedded female sand flea in the epidermis and the reaction of the host, and should be done according to the Fortaleza classification (Eisele et al. 2003, Parasitol Res, doi: 10.1007/s00436-002-0817-y). The clinical findings used as diagnostic criteria by the authors are a mixture of different parasitic stages, secondary complications (“surrounding tissue necrosis”) and manipulation of lesions by the patient or a caregiver (“crater-like score”). “Circular wounds in the keratin layer“ do not exist. The authors probably mean a circular impression in the epidermis corresponding to stage IV of the Fortaleza classification. Hence, the diagnosis of tungiasis was biased and will have resulted in an unknown number of false-positive and false-negative diagnoses and, by consequence, to an over-or-under-estimate of prevalence, respectively.

Line 293-296: Section belongs to Methods.

Line 407-408: The authors correctly state that the prevalence/incidence of scabies and tungiasis are influenced by the season of the year. For tungiasis the difference in prevalence between the end of the rainy season and the end of the dry season maybe up to 80 percent. Although the authors acknowledge this in line 423 they do not discuss the impact this will have on risk-factors which themselves are influenced by the season. For instance, in many endemic areas water supply is restricted in the dry season, and children may not be able to wash their feet even if soap is available. 

Line 423: Prevalence is not a rate but a proportion

Line 428: Prevalences between settings can only be compared if the studies were performed at the same season of the year.

Line 443-445: Change to “Our results similarly show that washing the feet daily with soap was a protective factor of both, tungiasis and tinea.

Line 488: Change to “We found significant associations between many individual hygiene-related factors and tinea, scabies and tungiasis.”

--------------------

PLOS authors have the option to publish the peer review history of their article (what does this mean?). If published, this will include your full peer review and any attached files.

If you choose “no”, your identity will remain anonymous but your review may still be made public.

**Do you want your identity to be public for this peer review?** For information about this choice, including consent withdrawal, please see our Privacy Policy.

Reviewer #1: Yes: Stéphane Ranque

Reviewer #2: No

Figure Files:

While revising your submission, please upload your figure files to the Preflight Analysis and Conversion Engine (PACE) digital diagnostic tool, https://pacev2.apexcovantage.com. PACE helps ensure that figures meet PLOS requirements. To use PACE, you must first register as a user. Then, login and navigate to the UPLOAD tab, where you will find detailed instructions on how to use the tool. If you encounter any issues or have any questions when using PACE, please email us at figures@plos.org.

Data Requirements:

Please note that, as a condition of publication, PLOS' data policy requires that you make available all data used to draw the conclusions outlined in your manuscript. Data must be deposited in an appropriate repository, included within the body of the manuscript, or uploaded as supporting information. This includes all numerical values that were used to generate graphs, histograms etc.. For an example see here: http://www.plosbiology.org/article/info%3Adoi%2F10.1371%2Fjournal.pbio.1001908#s5.

Reproducibility:

To enhance the reproducibility of your results, we recommend that you deposit your laboratory protocols in protocols.io, where a protocol can be assigned its own identifier (DOI) such that it can be cited independently in the future. Additionally, PLOS ONE offers an option to publish peer-reviewed clinical study protocols. Read more information on sharing protocols at https://plos.org/protocols?utm_medium=editorial-email&utm_source=authorletters&utm_campaign=protocols

References

Please review your reference list to ensure that it is complete and correct. If you have cited papers that have been retracted, please include the rationale for doing so in the manuscript text, or remove these references and replace them with relevant current references. Any changes to the reference list should be mentioned in the rebuttal letter that accompanies your revised manuscript. If you need to cite a retracted article, indicate the article's retracted status in the References list and also include a citation and full reference for the retraction notice.

# Author comment: Author response to Decision Letter 0

**DOI:** 10.1371/journal.pntd.0009816.r002

3 Sep 2021

Attachment Submitted filename: Response to Reviewers.docx Click here for additional data file.

# Peer review: Decision Letter 1

**DOI:** 10.1371/journal.pntd.0009816.r003

**Authors:** Bruce Y. Lee, Uwem Friday Ekpo

16 Sep 2021

Dear Mrs Amare,

We are pleased to inform you that your manuscript 'Risk factors for scabies, tungiasis, and tinea infections among schoolchildren in southern Ethiopia: A cross-sectional Bayesian multilevel model' has been provisionally accepted for publication in PLOS Neglected Tropical Diseases.

Before your manuscript can be formally accepted you will need to complete some formatting changes, which you will receive in a follow up email. A member of our team will be in touch with a set of requests.

Please note that your manuscript will not be scheduled for publication until you have made the required changes, so a swift response is appreciated.

IMPORTANT: The editorial review process is now complete. PLOS will only permit corrections to spelling, formatting or significant scientific errors from this point onwards. Requests for major changes, or any which affect the scientific understanding of your work, will cause delays to the publication date of your manuscript.

Should you, your institution's press office or the journal office choose to press release your paper, you will automatically be opted out of early publication. We ask that you notify us now if you or your institution is planning to press release the article. All press must be co-ordinated with PLOS.

Thank you again for supporting Open Access publishing; we are looking forward to publishing your work in PLOS Neglected Tropical Diseases.

Best regards,

Uwem Friday Ekpo, PhD

Associate Editor

PLOS Neglected Tropical Diseases

Bruce Lee

Deputy Editor

PLOS Neglected Tropical Diseases

***********************************************************

Reviewer's Responses to Questions

**Key Review Criteria Required for Acceptance?**

As you describe the new analyses required for acceptance, please consider the following:

**Methods**

-Are the objectives of the study clearly articulated with a clear testable hypothesis stated?

-Is the study design appropriate to address the stated objectives?

-Is the population clearly described and appropriate for the hypothesis being tested?

-Is the sample size sufficient to ensure adequate power to address the hypothesis being tested?

-Were correct statistical analysis used to support conclusions?

-Are there concerns about ethical or regulatory requirements being met?

Reviewer #1: (No Response)

**********

**Results**

-Does the analysis presented match the analysis plan?

-Are the results clearly and completely presented?

-Are the figures (Tables, Images) of sufficient quality for clarity?

Reviewer #1: (No Response)

**********

**Conclusions**

-Are the conclusions supported by the data presented?

-Are the limitations of analysis clearly described?

-Do the authors discuss how these data can be helpful to advance our understanding of the topic under study?

-Is public health relevance addressed?

Reviewer #1: (No Response)

**********

**Editorial and Data Presentation Modifications?**

Use this section for editorial suggestions as well as relatively minor modifications of existing data that would enhance clarity. If the only modifications needed are minor and/or editorial, you may wish to recommend “Minor Revision” or “Accept”.

Reviewer #1: (No Response)

**********

**Summary and General Comments**

Use this section to provide overall comments, discuss strengths/weaknesses of the study, novelty, significance, general execution and scholarship. You may also include additional comments for the author, including concerns about dual publication, research ethics, or publication ethics. If requesting major revision, please articulate the new experiments that are needed.

Reviewer #1: (No Response)

**********

PLOS authors have the option to publish the peer review history of their article (what does this mean?). If published, this will include your full peer review and any attached files.

If you choose “no”, your identity will remain anonymous but your review may still be made public.

**Do you want your identity to be public for this peer review?** For information about this choice, including consent withdrawal, please see our Privacy Policy.

Reviewer #1: **Yes: **Stéphane Ranque

# Editor report: Acceptance letter

**DOI:** 10.1371/journal.pntd.0009816.r004

**Authors:** Bruce Y. Lee, Uwem Friday Ekpo

21 Sep 2021

Dear Mrs Amare,

We are delighted to inform you that your manuscript, "Risk factors for scabies, tungiasis, and tinea infections among schoolchildren in southern Ethiopia: A cross-sectional Bayesian multilevel model," has been formally accepted for publication in PLOS Neglected Tropical Diseases.

We have now passed your article onto the PLOS Production Department who will complete the rest of the publication process. All authors will receive a confirmation email upon publication.

The corresponding author will soon be receiving a typeset proof for review, to ensure errors have not been introduced during production. Please review the PDF proof of your manuscript carefully, as this is the last chance to correct any scientific or type-setting errors. Please note that major changes, or those which affect the scientific understanding of the work, will likely cause delays to the publication date of your manuscript. Note: Proofs for Front Matter articles (Editorial, Viewpoint, Symposium, Review, etc...) are generated on a different schedule and may not be made available as quickly.

Soon after your final files are uploaded, the early version of your manuscript will be published online unless you opted out of this process. The date of the early version will be your article's publication date. The final article will be published to the same URL, and all versions of the paper will be accessible to readers.

Thank you again for supporting open-access publishing; we are looking forward to publishing your work in PLOS Neglected Tropical Diseases. 

Best regards,

Shaden Kamhawi

co-Editor-in-Chief

PLOS Neglected Tropical Diseases

Paul Brindley

co-Editor-in-Chief

PLOS Neglected Tropical Diseases
